# Multi-level language deficits in behavioural frontotemporal degeneration and related disorders

**DOI:** 10.1093/braincomms/fcag116

**Published:** 2026-04-01

**Authors:** Agnès Denève, Aurore Mahut Dubos, Thi Mai Tran, Antoine Renard, Coline Carpentier, Nathalie Forestier, Grégory Kuchcinski, Renaud Lopes, Ali Amad, Thibaud Lebouvier, Maxime Bertoux

**Affiliations:** Lille Neuroscience & Cognition, Inserm U1172, Univ Lille, CHU Lille, Lille F-59000, France; Lille Neuroscience & Cognition, Inserm U1172, Univ Lille, CHU Lille, Lille F-59000, France; Lille Neuroscience & Cognition, Inserm U1172, Univ Lille, CHU Lille, Lille F-59000, France; Liège Unité PsyNCog, Université de Liège, Liège 4000, Belgium; Lille Neuroscience & Cognition, Inserm U1172, Univ Lille, CHU Lille, Lille F-59000, France; Lille Neuroscience & Cognition, Inserm U1172, Univ Lille, CHU Lille, Lille F-59000, France; Lille Neuroscience & Cognition, Inserm U1172, Univ Lille, CHU Lille, Lille F-59000, France; Department of Nuclear Medicine, CHU Lille, Lille F-59000, France; Department of Neuroradiology, CHU Lille, Lille F-59000, France; Lille Neuroscience & Cognition, Inserm U1172, Univ Lille, CHU Lille, Lille F-59000, France; Department of Nuclear Medicine, CHU Lille, Lille F-59000, France; CNRS, INSERM, CHU Lille, Institut Pasteur de Lille, Univ. Lille, US 41 - UAR 2014 - PLBS, Lille F-59000, France; Lille Neuroscience & Cognition, Inserm U1172, Univ Lille, CHU Lille, Lille F-59000, France; Department of Psychiatry, CHU Lille, Lille F-59000, France; Lille Neuroscience & Cognition, Inserm U1172, Univ Lille, CHU Lille, Lille F-59000, France; Lille Neuroscience & Cognition, Inserm U1172, Univ Lille, CHU Lille, Lille F-59000, France

**Keywords:** frontotemporal degeneration, psychiatric disorders, major depressive disorder, bipolar disorder, fluency

## Abstract

Language is essential to social communication. Its complexity and hierarchical organization, from low-level operations to high-order integrative processes, may provide valuable diagnostic insights in neurodegeneration beyond classical aphasia syndromes. However, systematic investigations in these conditions, particularly in behavioural variant frontotemporal degeneration (bvFTD), remain scarce. To refine differential diagnosis, an exhaustive characterization of language functions is required.

We systematically compared multi-level language functioning across bvFTD, Alzheimer’s disease (AD) and primary psychiatric disorders (PPD), within an integrative neurolinguistic framework distinguishing lexical, syntactic and discursive levels, together with a cross-modal transposition/transcoding dimension. A total of 85 patients (including 34 with bvFTD, 30 with AD, 21 with PPD) and 40 matched healthy controls underwent an extensive language assessment using the GREMOTs battery. Composite, quantitative as well as qualitative indices were computed for each linguistic level. Structural MRI data were analysed using voxel-based morphometry (*P* < 0.05 corrected for multiple comparisons).

All clinical groups exhibited lexical impairments relative to controls, with bvFTD presenting the most severe and widespread deficits across fluency, naming and comprehension (partial eta-squared, *ηp*^2^, ranging from 0.18 to 0.49). AD and PPD showed milder lexical inefficiencies (*ηp*^2^ = 0.11–0.39 and *ηp*^2^ = 0.07–0.29, respectively). Syntactic processing was also more impaired in bvFTD (*ηp*^2^ = 0.03–0.27) than in AD and PPD (*ηp*^2^ = 0.02–0.07 and *ηp*^2^ = 0.00–0.15, respectively). At the discourse-level, bvFTD displayed key deficits (*ηp*^2^ = 0.05–0.29), with pervasive pragmatic breakdowns whereas AD and PPD showed milder integrative deficits with preserved global coherence (*ηp*^2^ = 0.00–0.17 and *ηp*^2^ = 0.02–0.13). Transcoding and transposing tasks revealed minor deficits, mainly in bvFTD (*ηp*^2^ = 0.01–0.25). A logistic regression identified that a subset of 12/23 tasks accurately classified 85.9% of bvFTD cases (sensitivity: 57.6%; specificity: 95.6%). The analysis of the types of responses (including errors) allowed to provide a more comprehensive group profiling. In bvFTD, the decrease of language performance related to widespread frontotemporal and posterior (including cerebellar) atrophy, whereas AD showed more restricted frontal and temporal involvement. PPD displayed smaller fronto-temporal, insular and precuneal associations.

In conclusion, these findings delineate a graded, multi-level linguistic profile across neurodegenerative and psychiatric conditions. bvFTD is mainly characterized by pervasive lexical and discursive–pragmatic impairments, alongside syntactic difficulties, while AD and PPD primarily show lexical inefficiencies with preserved syntax. Convergent neural evidence supports a distributed network model of language integrating frontal–insular control and temporal semantic systems. Embedding such multi-level assessments into clinical practice could enhance diagnostic precision and provide valid cognitive endpoints for future trials.

## Teaser

Denève *et al*. report that detailed language testing distinguishes behavioural frontotemporal degeneration from AD and PPD, highlighting the clinical value of multi-level language assessment. Patients with behavioural frontotemporal degeneration show widespread word, sentence and discursive impairments linked to brain atrophy, whereas the others show milder language changes.

## Graphical Abstract

**Graphical Abstract:**
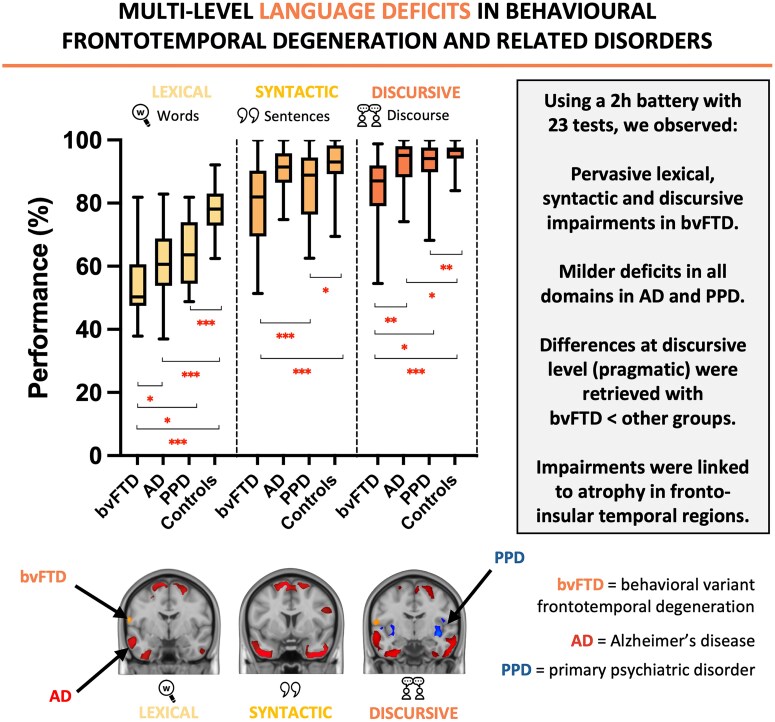

## Introduction

Language has long been a central focus in clinical neurosciences, first through the study of aphasia following focal brain lesions, and more recently, through its pivotal role in the diagnosis of primary progressive aphasia.^1^ Due to the complexity of its neural and cognitive architecture, as well as the considerable cross-cultural and cross-linguistic variability observed worldwide, language is generally regarded as an autonomous neurocognitive domain.^2^ Accordingly, it has been predominantly investigated within specialized fields such as neurolinguistics and psycholinguistics. From an epistemological standpoint, language has thus been conventionally treated as distinct from social cognition (as formalized in the DSM-5).^3^ Yet, this separation remains largely artificial: language is inherently embedded in social interactions and cannot be disentangled from the pragmatic and communicative contexts in which it is used.^4-6^

Language is indeed a cornerstone of social interactions, enabling individuals to convey intentions, express emotions, maintain relationships and navigate social norms.^4,7,8^ When disrupted, linguistic impairments compromise not only communication but also social engagement, social understanding and autonomy.^8^ Beyond classical aphasic syndromes,^1^ language dysfunction may therefore represent a key pathway to altered social behaviour. In this regard, language constitutes a particularly relevant domain to explore in non-aphasic conditions such as behavioural variant frontotemporal dementia (bvFTD) and its main differentials, including Alzheimer’s disease (AD) and late onset primary psychiatric disorders (PPD).^9^ Differentiating these conditions in clinical practice remains highly challenging.^10-12^ Core manifestations of bvFTD, such as disinhibition, apathy, reduced empathy and executive dysfunctions, often overlap with psychiatric symptoms,^13^ while both bvFTD and AD can also present with substantial memory and executive deficits.^14-16^ Such clinical overlap contributes to frequent diagnostic errors, which heighten family burden, prolong uncertainty and delay appropriate management.^17^ Although biomarkers have greatly improved diagnostic accuracy,^18^ they cannot be systematically obtained. Strengthening clinical approaches to differential diagnosis therefore remains a critical priority. Because language relies on multiple interacting cognitive processes supported by distributed neural networks^19,20^ and serves as the primary vehicle for social interactions,^4^ it represents a strategic domain through which to refine the differential diagnosis of these overlapping conditions.^21^

Language impairments in bvFTD typically manifest as reduced speech output, stereotyped or repetitive utterances and pragmatic disturbances, with the most consistent deficits involving lexico-semantics, orthography and prosody, while motor speech, grammar and phonology are relatively preserved.^22,23^ In AD, early stages are mainly characterized by lexico-semantic decline, lexical retrieval difficulties, reduced verbal fluency and pragmatic deficits, whereas syntax and phonology remain relatively preserved, although subtle phonological alterations may appear early.^24,25^ Although they are an integral part of psychiatric symptomatology, with distinct patterns emerging across disorders, language abnormalities have been less systematically explored in psychiatry. In major depressive disorder (MDD), they are often considered secondary to executive dysfunction, and include pragmatic and syntactic disturbances, such as shortened sentences due to omitted structural elements.^26,27^ In bipolar disorders (BD), linguistic alterations vary with mood state, ranging from pressured and tangential speech during mania to slowed, monotonous output with reduced lexical richness during depression, while discourse-level disturbances in organization, coherence and cohesion are observed across all phases, including euthymia.^28,29^ In schizophrenia, language is frequently and more profoundly affected, encompassing pragmatic impairments, reduced syntactic complexity and discourse incoherence, often accompanied by derailment, tangentiality, neologisms and paraphasia, despite overall preserved fluency.^30,31^

Most direct comparisons between bvFTD and AD have reported no significant group differences in language performance,^22,32-35^ although subtle distinctions may emerge through connected speech analysis.^36^ By contrast, direct comparisons between bvFTD and psychiatric disorders remain scarce and have mainly focused on fluency and naming, with few exceptions (e.g. on prosody).^37^ Studies comparing bvFTD with MDD or BD found no differences in semantic or phonemic fluency,^38,39^ whereas comparisons with schizophrenia revealed reduced semantic fluency in bvFTD, with inconsistent results for phonemic fluency and naming.^40,41^ To enhance statistical power, some studies grouped patients with heterogeneous psychiatric diagnoses, an efficient but confounding strategy.^13,42^ Under such conditions, bvFTD tends to show poorer performance on naming and verbal reasoning,^41^ yet findings remain inconsistent across fluency tasks.^41-43^ A more refined analysis of fluency, considering qualitative indices such as clustering, switching, or word frequency, could provide greater diagnostic sensitivity by capturing distinct contributions of semantic, executive and lexical processes.^44,45^ However no study has systematically compared these qualitative indicators across diseases.

Overall, language impairments in bvFTD are thus increasingly recognized,^22^ yet no study to date has provided a comprehensive and systematic characterization of this domain in this condition. Moreover, although previous findings point to substantial overlaps in language profiles across bvFTD, AD and PPD, no large-scale study has contrasted these groups across a broad range of linguistic processes. Finally, it remains unclear whether the similarities observed in language performance across these conditions reflect shared or distinct underlying neural mechanisms. Identifying the neural correlates of these deficits is therefore essential to disentangle disease-specific from transdiagnostic patterns.

The present study aims to fill these gaps by comparing quantitative and qualitative aspects of language performances across bvFTD, AD and PPD, using a specialized battery comprising 23 tasks. To this end, we adopted a neurolinguistic framework that decomposes language into three hierarchical levels: lexical, syntactic and discursive, together with a cross-cutting transposition/transcoding dimension. This framework, grounded in contemporary models of language architecture^2,20^ captures the progression from basic word retrieval and combinatorial operations to higher-order discourse integration, while the transcoding dimension accounts for cross-modal conversion processes between oral and written forms. Such a multi-level approach enables a fine-grained, modality-aware characterization of linguistic impairments across diseases. We hypothesized that patients with bvFTD would exhibit greater and more pervasive deficits than the other groups, with particularly marked impairments in higher-level, quintessentially social (i.e. discursive/pragmatic) abilities. Qualitative indices derived from tasks’ performance were examined to further elucidate the mechanisms underlying language disturbances. Structural imaging analyses were conducted to identify the neuroanatomical correlates of these linguistic dimensions.

## Materials and methods

### Participants

Patients were seen from 2016 to 2023 at the Lille memory clinic, national expert centre for rare and early degenerative diseases. Inclusion criteria required (i) a minimum clinical follow-up of 24 months (mean = 6.06 years), with at least two exhaustive neuropsychological (including 1 language assessment) and two MRI examinations at different time points to ensure diagnostic accuracy; (ii) French as native language; and (iii) review of each case by an interdisciplinary expert team of neurologists, psychiatrists, speech-therapists and neuropsychologists. Details of patients’ selection process are available in Supplementary Material 1.1.

From an initial group of 503 patients, 34 patients with probable bvFTD, 30 patients with typical AD, and 21 patients with a PPD (including *N* = 16 with MDD, *N* = 4 with BD and *N* = 1 with schizoaffective disorder) were selected, fulfilling diagnostic criteria for bvFTD,^46^ typical AD,^47^ and PPD,^3^ respectively. Among them, 50.0% had their diagnosis supported by abnormal cerebrospinal fluid levels of phospho-tau, total-tau, and beta-amyloid in the AD group, whereas 26.47% in the bvFTD group and 47.62% in the PPD group had a clinical diagnosis supported by the absence of an AD biomarker profile.

Forty healthy control participants matched to the bvFTD group for age, sex and education were also included. Control participants had no cognitive or psychiatric complaints or history, and normal cognitive efficiency as measured by the Mini-Mental State Examination (MMSE). The inclusion of all participants was covered with Lille University Hospital ethics approval (CHU-MR004-DEC25-151).

### Procedure

#### Multi-level quantitative language assessment

All participants were assessed in French by expert speech-therapists with the GREMOTs,^48^ a standardized computerized battery developed to assess language processes in neurodegenerative disorders. Details of the battery tasks are provided in Table 1. They addressed the three neurolinguistic hierarchical levels, i.e. lexical, syntactic and discursive, as well as a fourth mixed dimension requiring transpositions or transcoding across modalities (auditory-to-verbal, oral-to-written and vice versa).

**Table 1:** Structure of the GREMOTs language assessment

Composite scores	Tasks	Types of qualitative indicators
**Lexical level**		
	Action (verbs) fluency	
	Semantic (fruits) fluency	Repetition, intrusions, clusters, switches, frequency
	Phonological (V) fluency	Repetition, intrusions, clusters, switches, frequency
	Object naming	Categories (living, non-living)
	Action naming	
	Famous people naming	Professions (politicians, artists), gender
	Oral comprehension	Categories (target, semantically related, far and unrelated)
	Written comprehension	Categories (target, semantically related, far and unrelated)
**Syntactic level**		
	Command execution	
	Sentence production	
	Sentence comprehension	
**Discursive level**		
	Anamnestic interview & spontaneous speech	Fluency, fluidity, lexicon, syntax, comprehension, informativity, prosody, intelligibility, pragmatism and attention
	Narrative speech	Lexicon, syntax, informativity, pragmatism, actions and narrative quality
	Written texts understanding	
**Transcoding/transposing dimension**		
	Repetition of words	
	Repetition of non-words	
	Repetition of sentences	
	Reading aloud words	
	Reading aloud non-words	
	Writing words under dictation	
	Writing non-words under dictation	
	Writing sentences under dictation	
	Automatic writing	

The lexical level was assessed using eight tasks. Fluency (action, semantic and phonological) tasks required participants to produce as many verbs, fruit names, or words beginning with *V* as possible within two minutes, probing lexical retrieval, semantic search and executive control. Naming tasks involved identifying 36 objects, 36 actions and 10 famous faces, assessing lexical access, verb and noun retrieval, and semantic-to-phonological mapping. Comprehension tasks required judging whether a spoken or written word matched a picture, thus assessing auditory and visual lexical access as well as semantic comprehension. The same items were used in naming and comprehension tasks, allowing direct comparison between production and understanding abilities.

The syntactic level was assessed using three tasks. Command execution required participants to perform six oral instructions of increasing length and complexity, with or without object manipulation, engaging syntactic comprehension, working memory and motor planning. Sentence production involved orally generating six sentences from words presented simultaneously in oral and written form, varying in syntactic and semantic complexity to assess grammatical encoding and lexical integration. Sentence comprehension used a 24-items sentence–picture matching task, where participants selected the image corresponding to a spoken and written sentence, probing syntactic parsing and sentence-meaning mapping.

The discursive level was assessed using three tasks. Connected and spontaneous speech was evaluated during an anamnestic interview. Narrative speech required participants to narrate a five-picture sequence (with the speech-therapists supposedly having no access to the pictures) and to invent the end. Text comprehension involved reading three texts of increasing length and choosing, among four options, the sentence that best completed each story, assessing reading comprehension, inference making and integration of textual coherence.

The transcoding/transposing dimension was assessed using nine tasks. Word and non-word repetition required to repeat familiar or invented items, probing phonological memory and acoustic–phonological conversion. Sentence repetition engaged grammatical and phonological encoding as well as verbal working memory. Reading aloud words and non-words assessed lexical and phonological reading routes, grapheme–phoneme conversion and phonological memory. Writing dictation involved transcribing orally presented words, non-words and sentences, evaluating lexico-semantic and phoneme–grapheme conversion processes, alongside grammatical encoding and syntactic planning. Finally, automatic writing required writing overlearned sequences (name, address, signature), indexing automatized orthographic retrieval.

For each task, examples were provided after instructions. Rigorous psycholinguistic controls were applied to ensure validity. Word frequency, length and concreteness (e.g. in comprehension or naming tasks), phonological complexity and object familiarity (e.g. in command execution) were systematically controlled. Simultaneous oral and written presentations (e.g. sentence production) minimized short-term memory demands. The spatial arrangement of targets and distractors was counterbalanced and presented vertically to limit perseverative errors and hemineglect effects. Complete details on design, theoretical rationale and control procedures are published.^48^

To compute composite scores, tasks’ subscores for each level and the transcoding/transposing dimension were first converted to percentages of maximum possible (POMP) based on maximum scores or maximum controls performance. Similar unit-weight was then ensured through averaging those POMP-derived values within a single score.

#### Multi-level qualitative language assessment

In several tasks of our battery, to go beyond overall performance scores, we identified qualitative indicators (subscores, types of response, types of error, etc.) that allowed a more refined interpretation of participants’ performance.

##### Naming—object and famous people

Living and non-living categories were distinguished within the object naming task and politician and gender categories within the famous people naming task.

##### Comprehension—oral and written

Each target was paired with three semantically distractors (related, far and unrelated). To score one point, participants had to respond ‘yes’ to the target and ‘no’ to all three distractors directly, without self-correction. Responses to each category were recorded.

##### Fluency—semantic and phonological

Responses from the semantic and phonological fluency tasks were analysed to assess qualitative fluency indicators. Audio recordings were manually transcribed, and repetition and intrusion errors were counted. Clustering metrics (number, size, mean size) and switches between semantic or phonemic subcategories were computed, as detailed in Supplementary Material 1.2 and Supplementary Table 1, and subsequently analysed in covariance with the total number of words evoked, since intrusions and repetitions are included in cluster and switch counts. Lexical frequency was also calculated for all appropriate responses and analysed in covariance with the number of correct words, as intrusions and repetitions were excluded from this measure.

##### Anamnestic interview

Speech therapists rated fluency, lexicon, syntax, informativeness, prosody, intelligibility, pragmatics and attention on a 0–5 scale.

##### Narrative speech

Narrative structure, lexical and syntactic complexity, informativeness and pragmatic abilities were rated.

#### Neuroimaging acquisition, pre-processing and harmonization

Details of MRI acquisition and preprocessing are provided in Supplementary Materials 1.2. The final sample included 22 bvFTD, 12 PPD and 15 AD patients with structural MRI. Briefly, T1-weighted images were brain-extracted, segmented and normalized to Montreal Neurological Institute space using FSL.^49^ A study-specific template was generated; grey matter maps were non-linearly registered, modulated and smoothed with a 4 mm FWHM Gaussian kernel.

To control scanner-related variability, the ComBat harmonization (neuroHarmonize) method removed site-specific additive and multiplicative effects while preserving biologically relevant variation. Voxel intensity values were first extracted for each participant and compiled into a participant × voxels matrix (from grey matter). Covariates of interest, including age, education and gender, were entered as variables to preserve. Scanner identity was treated as a batch variable. Harmonized images were then reconstructed in standard space and submitted to group-level statistical analyses.

### Statistical analysis

#### Demographics and cognitive scores

Statistical analyses were conducted using IBM SPSS (30.0.0.0). Chi-square (*χ*2) and Student tests were used to analyse demographic data, after log-transformation when needed. Covariance analyses were then performed (ANCOVAs adjusted for age, education level) to compare quantitative and qualitative language data across groups for each task. We calculated partial eta-squared (*ηp*2) values to estimate effect sizes and 95% confidence interval (95% CI) to indicate the precision of the estimates. A backward stepwise logistic regression (likelihood ratio method) was performed to identify the set of scores (among the 23 tasks) best distinguishing the bvFTD group.

#### Voxel-based morphometry (VBM)

VBM analyses were performed with FSL. Separate voxel-wise general linear models (GLMs) were run for each group and composite score to test associations between reduced linguistic performance and decreased grey matter density. Each analysis was tested with 5000 permutations and familywise error correction (FWE, *P* < 0.05). Clusterwise significance was determined with threshold-free cluster enhancement (TFCE),^49,50^ and only clusters >100 voxels are reported. Age, education and sex were included as nuisance covariates.

Given the hierarchical organization of linguistic levels, additional GLMs were computed including lower-level composite scores as covariates. The lexical score was controlled for syntactic analyses, and both lexical and syntactic scores were controlled for discursive analyses. For the transposing/transcoding dimension, the lexical score was included as a covariate. This approach isolated correlations specific to each linguistic level and to the transcoding dimension.

### Data availability

Access to anonymized data could be granted on demand in accordance with ethical guidelines and subject to a data-sharing agreement with Lille University Hospital. Commercial use is not permitted. Scripts written to facilitate the extraction of qualitative indicators are freely available online (https://osf.io/bychj).^51^

## Results

### Demographics

Characteristics of patients and control samples are presented in Table 2. The AD group was older compared to PPD (*t* = 5.616, *P* < 0.001; 95% CI [7.971, 16.855]), bvFTD (*t* = 2.050, *P* = 0.045; [0.111, 8.719]) and controls (*t* = 3.556, *P* < 0.001; [3.467, 12.335]), with bvFTD group being older than the PPD group (*t* = 3.483, *P* = 0.001; [3.392, 12.605]). No differences were observed for PPD (*t* = −1.856, *P* = 0.068; [−9.378, 0.353]) and bvFTD (*t* = 1.582, *P* = 0.118; [−0.907, 7.877]) compared to controls. Moreover, no differences were obtained between groups for sex (bvFTD versus PPD: *χ*^2^(1, *N* = 55) = 0.044, *P* = 0.834; *t* = 0.206, *P* = 0.838; [−0.245, 0.301], AD versus bvFTD: *χ*^2^(1, *N* = 64) = 0.855, *P* = 0.355; *t* = −0.916, *P* = 0.363; [−0.362, 0.135], AD versus PPD: *χ*^2^(1, *N* = 51) = 0.370, *P* = 0.543; *t* = −0.598, *P* = 0.552; [−0.374, 0.202], AD versus controls: *χ*^2^(1, *N* = 70) = 0.121, *P* = 0.728; *t* = −0.343, *P* = 0.733; [−0.284, 0.201], bvFTD versus controls: *χ*^2^(1, *N* = 74) = 0.400, *P* = 0.527; *t* = 0.626, *P* = 0.533; [−0.157, 0.302], PPD versus controls: *χ*^2^(1, *N* = 61) = 0.110, *P* = 0.740; *t* = 0.327, *P* = 0.745; [−0.225, 0.313]), nor for education (bvFTD versus PPD: *t* = 1.856, *P* = 0.069; [−0.127, 3.295], AD versus bvFTD: *t* = 0.644, *P* = 0.522; [−1.246, 2.430], AD versus controls: *t* = 1.800, *P* = 0.076; [−0.164, 3.181], bvFTD versus controls: *t* = 1.218, *P* = 0.227; [−0.583, 2.415], PPD versus controls: *t*=−0.865, *P* = 0.391; [−2.213, 0.877]), with the exception of AD group having a higher level of education compared to PPD (*t* = 2.429, *P* = 0.019; [0.375, 3.978]).

**Table 2:** Demographics of clinical and control groups

	Mean ± SD/frequency			
Variable	bvFTD	AD	PPD	Controls
*N*	34	30	21	40
Age (years)	67.8^a^ ± 8.92	72.3^b^ ± 8.21	59.8^a^ ± 7.08	64.3 ± 9.87
**Sex**				
Male (*n,* %)	22, 65.0%	16, 53.0%	13, 62.0%	23, 59.0%
Female (*n*, %)	12, 35.0%	14, 47.0%	8, 38.0%	17, 43.0%
**Handedness**				
Right-handed (*n*, %)	33, 97.1%	26, 86.7%	18, 85.7%	37, 92.5%
Left-handed (*n*, %)	0, 0.0%	3, 10.0%	2, 9.5%	2, 5.0%
Ambidextrous (*n*, %)	1, 2.9%	0, 0.0%	1, 4.8%	1, 2.5%
Forced right-hander (*n*, %)	0, 0.0%	1, 3.3%	0, 0.0%	0, 0.0%
Education (years)	11.4 ± 3.40	12.0 ± 3.95	9.86^b^ ± 2.43	10.5 ± 3.06
MMSE (score/30)	23.2^a^ ± 4.05	23.7^a^ ± 4.66	23.7^a^ ± 3.85	28.6 ± 1.20

SD = standard deviation; bvFTD = behavioural variant frontotemporal degeneration; PPD = primary psychiatric disorder; AD = Alzheimer’s disease; *N* = sample size; MMSE = mini-mental state examination.

^a^versus AD (*P* < 0.05).

^b^versus controls (*P* < 0.05).

Compared to controls, the MMSE score was lower for the bvFTD (*t* = −6.336, *P* < 0.001; [−0.295, −0.151]), PPD (*t* = −4.858, *P* < 0.001; [−0.288, −0.114]) and AD groups (*t* = −4.418, *P* < 0.001; [−0.312, −0.114]), with no differences between those groups (AD versus bvFTD: *t* = 0.183, *P* = 0.855; [−0.127, 0.105], AD versus PPD: *t* = −0.171, *P* = 0.865; [−0.123, 0.146], bvFTD versus PPD: *t* = −0.405, *P* = 0.687; [−0.131, 0.087]).

### Multi-level quantitative language assessment

**Figure 1:**
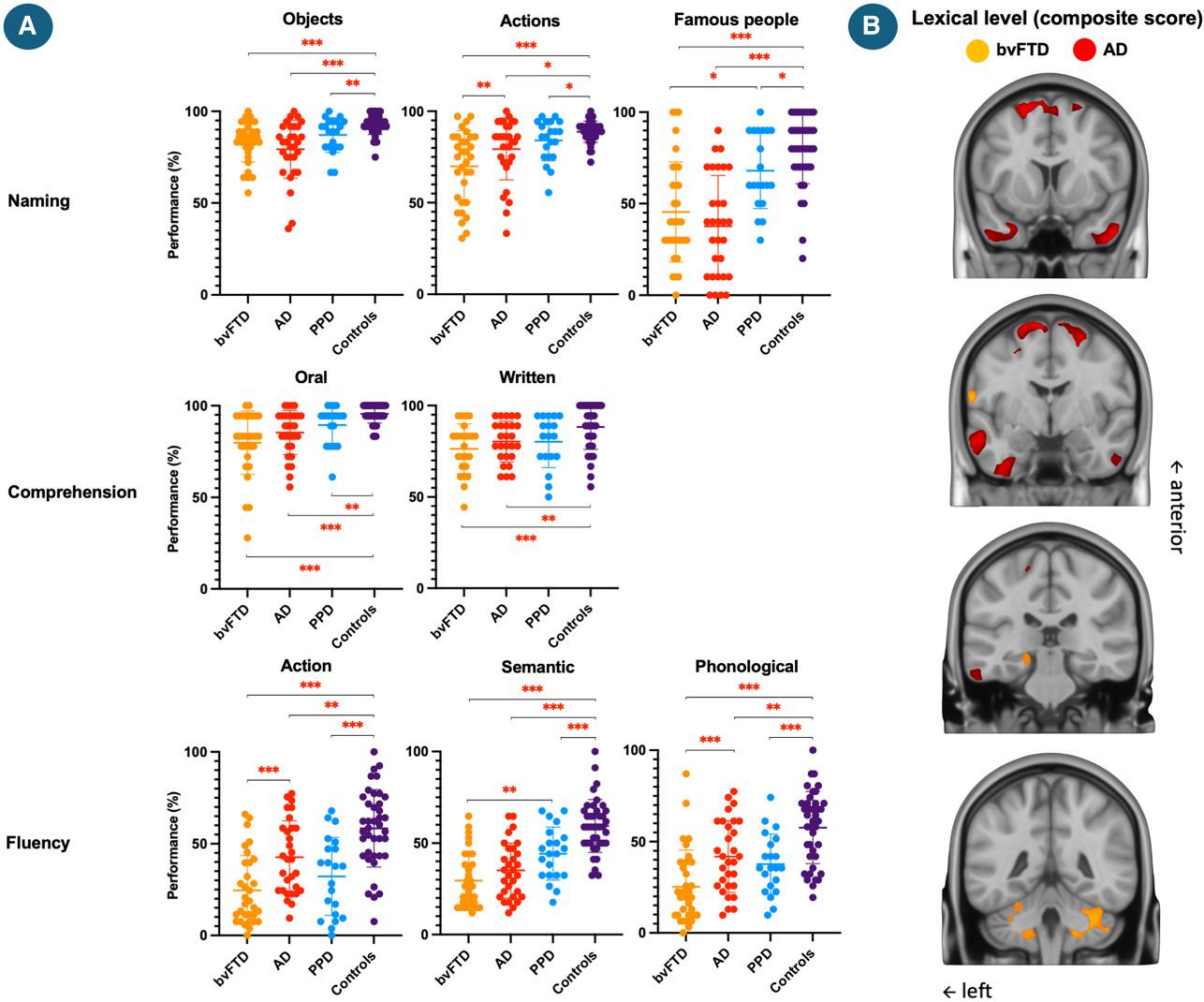
**Participants’ performance at the tasks composing the lexical level (A) and correlation of the lexical level composite score with grey matter density in the bvFTD, AD and PPD groups (B).** Abbreviations: bvFTD = behavioural variant frontotemporal degeneration; AD = Alzheimer’s disease; PPD = primary psychiatric disorder; % = percentage. A: post-hoc comparisons were performed following an ANCOVA, with age and education level included as covariates, *P < 0.05; **P < 0.01; ***P < 0.001, each data point represents an individual participant, *N*_bvFTD_ = 34, *N*_AD_ = 30, *N*_PPD_ = 21, *N*_controls_ = 40; B: voxel-based morphometry analyses tested associations between language scores and grey matter, controlling for age, education, and sex. Significance was assessed with 5000 permutations, familywise error correction (*P* < 0.05), and threshold-free cluster enhancement, *N*_bvFTD_ = 22, *N*_AD_ = 15, *N*_PPD_ = 12.

**Figure 2:**
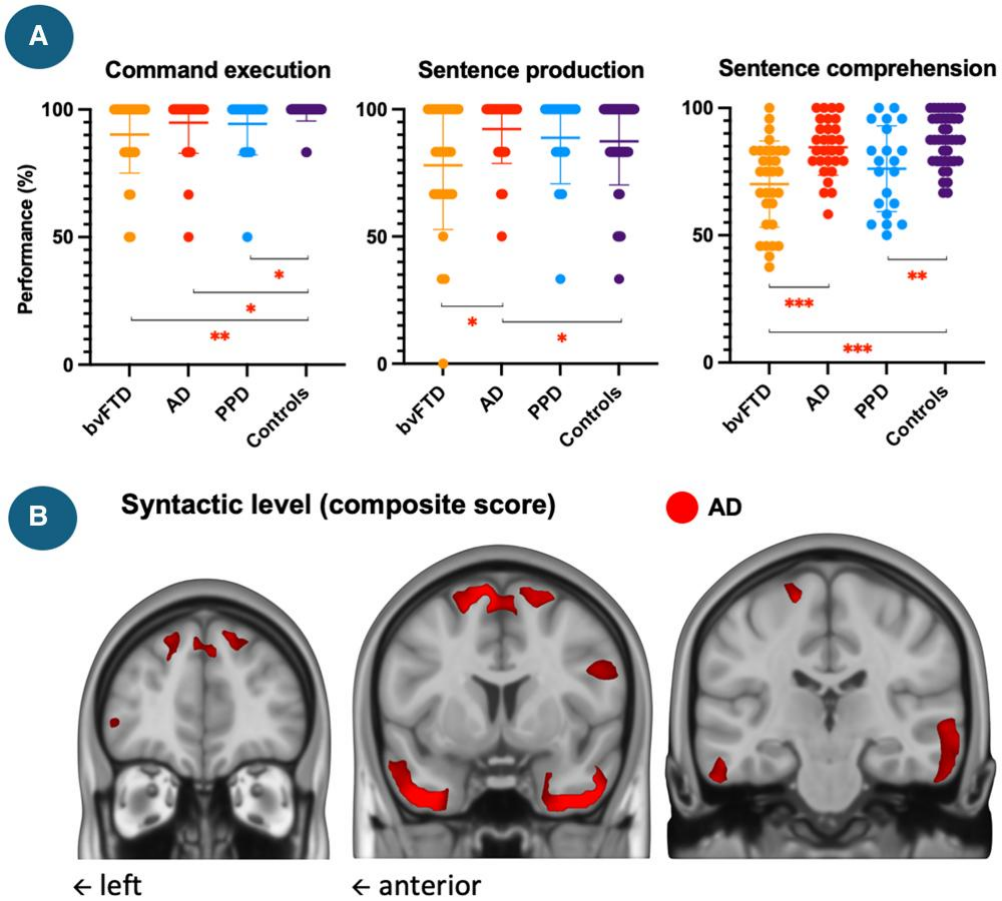
**Participants’ performance at the tasks composing the syntactic level (A) and correlation of the syntactic level composite score with grey matter density in the bvFTD, AD and PPD groups (B).** Abbreviations: bvFTD = behavioural variant frontotemporal degeneration; AD = Alzheimer’s disease; PPD = primary psychiatric disorder; % = percentage. A: post-hoc comparisons were performed following an ANCOVA, with age and education level included as covariates, *P < 0.05; **P < 0.01; ***P < 0.001, each data point represents an individual participant, *N*_bvFTD_ = 34, *N*_AD_ = 30, *N*_PPD_ = 21, *N*_controls_ = 40; B: voxel-based morphometry analyses tested associations between language scores and grey matter, controlling for age, education, sex, and lower-level language scores (lexical). Significance was assessed with 5000 permutations, familywise error correction (*P* < 0.05), and threshold-free cluster enhancement, *N*_bvFTD_ = 22, *N*_AD_ = 15, *N*_PPD_ = 12.

**Figure 3:**
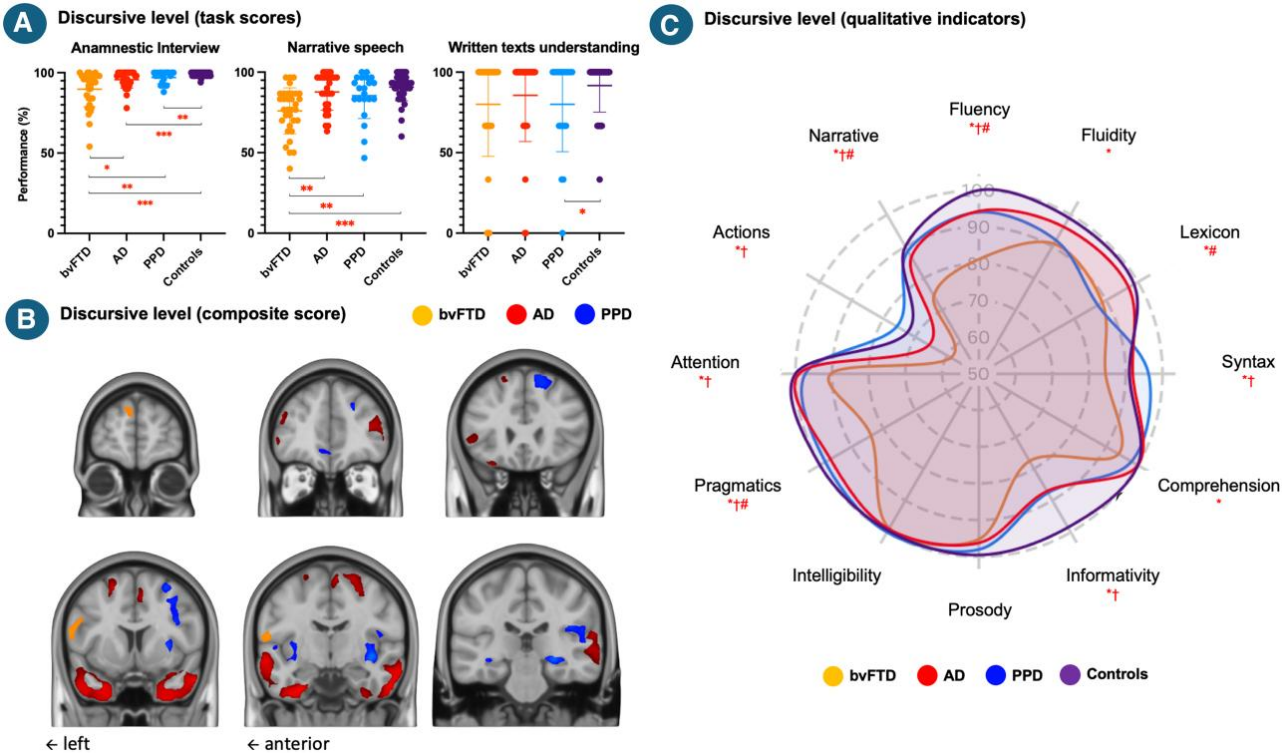
**Participants’ performance at the tasks composing the discursive level (A), correlations of the discursive level composite score with grey matter density in the bvFTD, AD and PPD groups (B) and qualitative indicators extracted from discursive tasks (C).** Abbreviations: bvFTD = behavioural variant frontotemporal degeneration; AD = Alzheimer’s disease; PPD = primary psychiatric disorder; % = percentage. A: post-hoc comparisons were performed following an ANCOVA, with age and education level included as covariates, *P < 0.05; **P < 0.01; ***P < 0.001, each data point represents an individual participant, *N*_bvFTD_ = 34, *N*_AD_ = 30, *N*_PPD_ = 21, *N*_controls_ = 40; B: voxel-based morphometry analyses tested associations between language scores and grey matter, controlling for age, education, sex, and lower-level language scores (lexical and syntactic). Significance was assessed with 5000 permutations, familywise error correction (*P* < 0.05), and threshold-free cluster enhancement, with *N*_bvFTD_ = 22, *N*_AD_ = 15, *N*_PPD_ = 12; C: post-hoc comparisons were performed following an ANCOVA, with age and education level included as covariates, *bvFTD versus controls (*P* < 0.05); ^#^bvFTD versus PPD (*P* < 0.05); ^†^bvFTD versus AD (*P* < 0.05).

**Figure 4:**
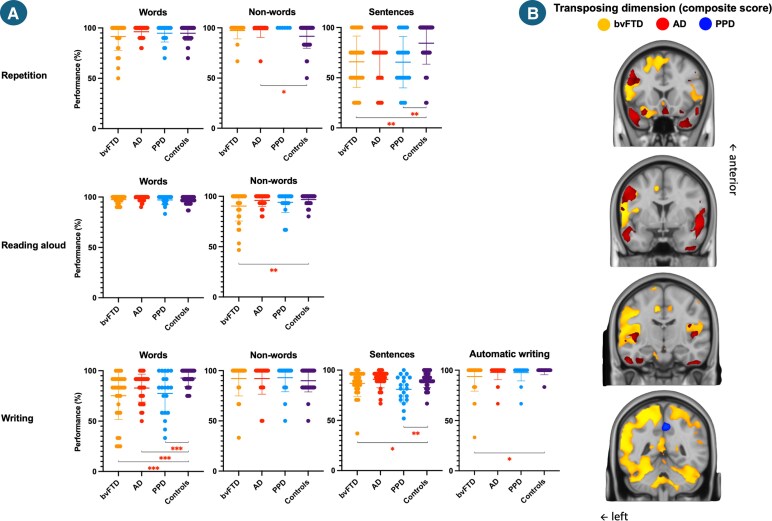
**Participants’ performance at the tasks composing the transposing/transcoding dimension (A) and correlation of the dimension’s composite score with grey matter density in the bvFTD, AD and PPD groups (B).** Abbreviations: bvFTD = behavioural variant frontotemporal degeneration; AD = Alzheimer’s disease; PPD = primary psychiatric disorder; % = percentage. A: post-hoc comparisons were performed following an ANCOVA, with age and education level included as covariates, *P < 0.05; **P < 0.01; ***P < 0.001, each data point represents an individual participant, *N*_bvFTD_ = 34, *N*_AD_ = 30, *N*_PPD_ = 21, *N*_controls_ = 40; B: voxel-based morphometry analyses tested associations between language scores and grey matter, controlling for age, education, sex, and lower-level language scores (lexical). Significance was assessed with 5000 permutations, familywise error correction (*P* < 0.05), and threshold-free cluster enhancement, *N*_bvFTD_ = 22, *N*_AD_ = 15, *N*_PPD_ = 12.

Groups’ performances for composite scores are reported in Table 3 and below. Due to space constraint, statistical comparisons for each task are only synthetized here but detailed in Supplementary Material 2.1, Supplementary Table 2 and presented in Figs 1–4.

**Table 3:** Quantitative language performance across clinical and control groups

	Mean ± SD			
Domains	bvFTD	AD	PPD	Controls
*N*	34	30	21	40
Lexical (%)	54.3^a,^b^,^c^^ ± 12.0	60.1^c^ ± 11.5	64.9^c^ ± 10.5	77.6 ± 7.42
Syntactic (%)	79.5^b,^c^^ ± 13.2	90.2 ± 7.18	86.2^c^ ± 10.8	91.4 ± 7.95
Discursive (%)	84.2^a,^b^,^c^^ ± 11.5	92.4^c^ ± 6.88	92.0^c^ ± 7.94	95.8 ± 3.55
Transcoding (%)	79.6^c^ ± 7.36	82.1 ± 4.37	80.4 ± 5.62	82.6 ± 4.45

Mean percentage of correct performance for the composite scores corresponding to each linguistic level and the cross-cutting dimension.

SD = standard deviation; bvFTD = behavioural variant frontotemporal degeneration; PPD = primary psychiatric disorder; AD = Alzheimer’s disease; *N* = sample size; % = percentage.

^a^versus PPD (*P* < 0.05).

^b^versus AD (*P* < 0.05).

^c^versus controls (*P* < 0.05).

For the lexical composite score, the bvFTD group demonstrated a lower performance compared to the PPD (*F* = 6.794, *P* = 0.012; *ηp*2 = 0.12; 95% CI [−17.053, −2.209]), AD (*F* = 5.837, *P* = 0.019; *ηp*2 = 0.09; [−13.369, −1.256]) and controls groups (*F* = 96.47, *P* < 0.001; *ηp*2 = 0.57; [−27.698, −18.346]), as did the AD (*F* = 46.59, *P* < 0.001; *ηp*2 = 0.41; [−20.390, −11.160]) and PPD groups (*F* = 36.74, *P* < 0.001; *ηp*2 = 0.39; [−16.620, −8.366]) compared to controls. No difference was retrieved between the AD and PPD groups (*F* = 0.000, *P* = 0.982; *ηp*2 = 0.00; [−8.037, 7.859]). As shown in Fig. 1, all clinical groups exhibited broad impairments across naming, comprehension and fluency tasks relative to controls, with bvFTD generally most affected.

Regarding the syntactic composite score, the bvFTD group demonstrated a lower performance compared to AD (*F* = 14.256, *P* < 0.001; *ηp*2 = 0.19; [−16.480, −5.063]) and controls groups (*F* = 18.653, *P* < 0.001; *ηp*2 = 0.21; [−16.326, −6.009]). The PPD group also had a lower performance compared to controls (*F* = 6.07, *P* = 0.017; *ηp*2 = 0.10; [−10.729, −1.109]). No differences were retrieved between the bvFTD and PPD groups (*F* = 2.680, *P* = 0.108; *ηp*2 = 0.05; [−14.570, 1.484]), nor between the AD and PPD groups (*F* = 1.49, *P* = 0.228; *ηp*2 = 0.03; [−2.391, 9.770]). Patients with AD did not differ from controls (*F* = 0.008, *P* = 0.931; *ηp*2 = 0.00; [−4.057, 3.718]). As shown in Fig. 2, across the tasks composing this level, the bvFTD group tended to be more impaired than the AD and controls groups, AD and PPD presenting intermediate deficits.

At the discursive level, the bvFTD group demonstrated a lower performance compared to the PPD (*F* = 6.876, *P* = 0.012; *ηp*2 = 0.12; [−15.346, −2.034]), AD (*F* = 9.745, *P* = 0.003; *ηp*2 = 0.14; [−13.006, −2.845]) and controls groups (*F* = 36.426, *P* < 0.001; *ηp*2 = 0.35; [−15.967, −8.034]). Patients with AD (*F* = 4.972, *P* = 0.029; *ηp*2 = 0.07; [−5.737, −0.317]) and PPD (*F* = 7.728, *P* = 0.007; *ηp*2 = 0.12; [−6.477, −1.053]) also had lower performance as compared to controls, with no differences being observed between them (*F* = 1.385, *P* = 0.245; *ηp*2 = 0.03; [−2.187, 8.355]). Across the tasks (Fig. 3), the bvFTD group showed the most pervasive impairments, performing worse than all other groups on the anamnestic interview and narrative speech. Written text understanding was relatively spared in bvFTD and AD, with a mild impairment in PPD.

Regarding the transcoding/transposing dimension, the bvFTD group had lower abilities compared to controls (*F* = 5.228, *P* = 0.025; *ηp*2 = 0.07; [−6.210, −0.423]). No other differences were observed between groups (bvFTD versus PPD: *F* = 1.157, *P* = 0.287; *ηp*2 = 0.02; [−6.597, 1.996], AD versus bvFTD: *F* = 1.535, *P* = 0.220; *ηp*2 = 0.02; [−1.212, 5.151], AD versus PPD: *F* = 1.193, *P* = 0.280; *ηp*2 = 0.02; [−1.627, 5.496], AD versus controls: *F* = 0.057, *P* = 0.811; *ηp*2 = 0.00; [−2.560, 2.011], PPD versus controls: *F* = 3.202, *P* = 0.079; *ηp*2 = 0.05; [−5.106, 0.287]). When examining tasks performance (Fig. 4), repetition and reading were largely preserved with only mild group differences, whereas writing under dictation was impaired in all clinical groups and sentence repetition deficits were evident in bvFTD and PPD; bvFTD also showed slight difficulties in automatic writing.

### Multi-level qualitative language assessment

Details of the statistical groups’ comparisons are available in Supplementary Material 2.2.

#### Lexical level

In the naming tasks (Supplementary Table 3), all patient groups demonstrated reduced performance for objects, actions and famous people. Patients with AD were the most impaired overall, except for action naming, in which bvFTD patients showed the greatest deficit. For object naming, all clinical groups obtained reduced performance on living and non-living objects without any differences between clinical groups. For famous people naming, all clinical groups demonstrated reduced performance for women and politicians, with subtle differences between groups. Performance was significantly worse in bvFTD compared to AD patients on action naming and compared to PPD patients on famous people naming.

For the fluency tasks, results are shown in Table 4 and detailed information are presented in Supplementary Material 2.2. All patient groups produced fewer correct words and showed reduced clustering and switching, with bvFTD being most impaired. Error patterns diverged: intrusions were more frequent in bvFTD and AD, repetitions in AD only. In semantic fluency, all groups generated fewer rare or very rare words, whereas in phonological fluency, this reduction was limited to bvFTD and PPD. Most group differences, however, disappeared after controlling for total word output.

**Table 4:** Qualitative data of lexico-semantic processing

	Mean ± SD			
Verbal fluency	bvFTD	AD	PPD	Controls
**Semantic**				
*N*	30	29	18	37
Number of words	12.3^a,^b^^ ± 5.28	15.4^b^ ± 5.28	15.6^b^ ± 4.46	21.8 ± 5.24
Repetitions	1.57 ± 2.08	2.79^b^ ± 2.70	0.89 ± 1.18	0.95 ± 1.20
Intrusions	0.67^b^ ± 1.27	0.59^b^ ± 0.78	0.44 ± 0.86	0.13 ± 0.35
*N*	30	29	16	37
Total cluster size	4.73^b^ ± 3.12	6.07^b^ ± 3.51	6.06^b^ ± 3.26	9.65 ± 3.63
Cluster number	3.27^b^ ± 2.12	4.03^b^ ± 1.99	4.19^b^ ± 1.68	5.92 ^±^ 1.62
Mean cluster size	1.46 ± 0.51	1.47 ± 0.59	1.39^a,b^ ± 0.27	1.63 ^±^ 0.40
Switch	6.57^a,^b^^ ± 3.05	8.55^b^ ± 3.17	8.44^b^ ± 2.42	11.0 ^±^ 2.19
*N*	30	29	18	37
Total frequency	116^b^ ± 47.2	124^b^ ± 59.7	139^b^ ± 58.3	170 ^±^ 49.6
Very frequent	0.03 ± 0.18	0.17 ± 0.38	0.17 ± 0.38	0.11 ^±^ 0.31
Frequent	1.00^c^ ± 0.59	0.83^b^ ± 0.38	0.89^b^ ± 0.47	1.03 ^±^ 0.29
Rare	3.07^b^ ± 1.48	3.07^b^ ± 1.33	3.33^b^ ± 1.33	4.97 ^±^ 1.61
Very rare	6.43^b^ ± 4.01	7.52^b^ ± 4.03	10.1^b^ ± 3.62	13.4 ^±^ 3.79
**Phonemic**				
*N*	31	30	17	38
Number of words	9.29^a,^b^^ ± 6.08	15.9 ± 7.26	12.1^b^ ± 5.48	19.0 ^±^ 6.18
Repetitions	0.81 ± 0.79	1.67^b^ ± 1.94	0.47 ± 0.62	0.47 ^±^ 0.72
Intrusions	0.48 ± 0.68	0.70 ± 1.29	0.23 ± 0.44	0.34 ^±^ 0.58
*N*	31	30	16	38
Total cluster size	3.00^a,^b^^ ± 3.63	5.03 ± 2.88	3.69^b^ ± 2.77	7.03 ^±^ 4.26
Cluster number	1.94^a,^b^^ ± 2.11	3.97 ± 2.43	2.19^b^ ± 1.42	4.66 ^±^ 2.36
Mean cluster size	1.09 ± 0.76	1.26 ± 0.54	1.73^a^ ± 0.70	1.40 ^±^ 0.65
Switch	5.32^a,^b^^ ± 3.31	10.2 ± 5.49	6.25^a,^b^^ ± 4.48	11.1 ^±^ 3.88
*N*	30	30	17	38
Total frequency	1714^a,^b^^ ± 2843	5105 ± 4814	4015 ± 3882	4759 ^±^ 5540
Very frequent	1.40^a,^b^^ ± 1.65	2.97 ± 2.17	2.53 ± 1.91	2.84 ^±^ 1.90
Frequent	1.13^a,^b^^ ± 1.07	2.00 ± 1.84	2.06 ± 1.82	2.16 ^±^ 1.65
Rare	1.57^a,^b^^ ± 1.01	2.93 ± 1.93	2.41 ± 1.42	3.53 ^±^ 1.74
Very rare	3.93^b^ ± 3.75	5.43^b^ ± 3.70	4.24^b^ ± 2.54	9.24 ^±^ 4.17

SD = standard deviation; bvFTD = behavioural variant frontotemporal degeneration; PPD = primary psychiatric disorder; AD = Alzheimer’s disease; *N* = sample size; % = percentage.

^a^versus AD (*P* < 0.05). Results adjusted for the number of evoked words (cluster and switch scores) and for the number of correct words (frequency scores) are provided in the Supplementary material.

^b^versus controls (*P* < 0.05).

^c^versus PPD (*P* < 0.05).

In the comprehension tasks, all patient groups exhibited reduced performance on both oral and written comprehension, except PPD, whose written comprehension remained preserved. For oral comprehension, patients with bvFTD and PPD produced a higher number of semantically related error types compared with controls. Patients with bvFTD also had a higher number of this type of errors compared to patients with AD. For written comprehension, a higher number of semantically far errors was retrieved in bvFTD as compared to controls, and a higher number of semantically unrelated errors as compared to PPD.

#### Discursive level

As shown in Fig. 3C, the bvFTD group was the most impaired (subscores for fluency, pragmatism and narrative being lower than for every other group), with AD and PPD also showing some deficits relative to controls, particularly in fluency, lexicon and informativity. Groups’ means and standard deviations are available in Supplementary Table 4 and Fig. 3C, and results are detailed in Supplementary Material 2.2.

### Diagnostic accuracy of language assessment

The logistic regression model identified (*χ*^2^(9) = 50.714, *P* < 0.001) anamnestic interview, written text understanding, object naming, action naming, famous people naming, semantic fluency, phonological fluency, command execution, sentence comprehension, sentence repetition, words writing and automatic writing as predictors. This set of 12 best predictors correctly classified 85.9% of participants (with 57.6% sensitivity and 95.6% specificity) as having bvFTD or not.

### VBM—multi-level intra-group correlations

All correlations presented were significant and positive (i.e. the lower the performance, the lower the density). Regional labels were derived from the Harvard-Oxford cortical structural atlas. Details of the imaging findings are presented in Supplementary Tables 5–7.

In bvFTD (Figs 1–4), the lexical composite correlated with grey matter density in the left precentral and parahippocampal gyri and bilateral posterior cerebellum. No significant effects emerged for the syntactic score. The discursive score correlated with clusters spanning left parietal–occipital and frontal (inferior and superior, including frontal pole) regions and a smaller right parietal cluster. The transcoding score showed an extensive bilateral (predominantly left) pattern involving frontopolar, inferior frontal, precentral, insular, temporal (superior/middle), parietal (postcentral), occipital (inferior/lingual) and cerebellar regions.

In AD, the lexical composite correlated with grey matter density in bilateral superior frontal gyri (including the supplementary motor cortex) and in bilateral temporal poles extending leftward into the posterior middle/inferior temporal and fusiform cortices. Syntactic correlations involved larger, more bilateral clusters within these same regions, extending to the right posterior temporal (middle/inferior) and fusiform cortices and the right precentral gyrus. Discursive correlations also encompassed both temporal lobes and smaller superior frontal areas, with additional bilateral (predominantly left) inferior frontal (pars triangularis/opercularis) clusters. The transcoding score was associated with bilateral frontopolar (paracingulate, subcallosal, ventromedial) and lateral frontal (middle, precentral, orbital) regions, mainly left-lateralized, as well as bilateral temporal poles extending to Heschl’s gyri and anterior insulae.

In PPD, no significant correlations emerged for the lexical or syntactic composites. The discursive score correlated with a large right-lateralized cluster spanning the superior, middle and precentral frontal gyri, insula, posterior superior/middle temporal and parahippocampal gyri, along with smaller clusters in the right lingual gyrus and left precentral, insular, posterior superior temporal and parahippocampal regions. The transcoding score correlated with midline posterior areas encompassing the bilateral precuneus and extending into the left posterior cingulate cortex.

In the additional GLMs controlling for lower-level linguistic processes, no significant effects were found for the syntactic composite in any group. For the discursive score, results replicated those of the primary analyses in AD and PPD but no longer reached significance in bvFTD. For the transcoding dimension, bvFTD patients showed correlations in the left temporoparietal junction, inferior parietal lobule, insula and inferior temporal–occipital regions (including the fusiform and inferior occipital gyri), while no significant clusters were observed in AD or PPD.

## Discussion

In this clinically well-characterized cohort, language functioning was examined across bvFTD, AD and PPD using a neurolinguistic hierarchical framework distinguishing lexical, syntactic and discursive levels, together with a cross-modal transposition/transcoding dimension. Three main results emerged: (i) all patient groups showed lexical impairments, with the largest deficits found in bvFTD; (ii) syntactic performance was relatively preserved except for a mild decrement in bvFTD; and (iii) discursive–pragmatic abilities were most affected in bvFTD. These results delineate a graded multi-level profile that distinguishes clinical groups and underscore the diagnostic value of language beyond classic aphasia syndromes.

At the lexical level, all patient groups were impaired relative to controls, but with distinct patterns reflecting specific linguistic profiles. The bvFTD group showed the most severe and generalized deficits, with markedly reduced fluency, naming and comprehension scores. These impairments were accompanied by increased semantic intrusions and reduced clustering, switching and lexical frequency in fluency tasks. In comprehension tasks, patients had access to target meanings but showed impaired discrimination of semantically related items. Whether this pattern indicates a mild degradation of semantic knowledge or could rather be interpreted as an impaired regulation of semantic access cannot directly be answered with our data. At the neural level, lexical performance correlated with grey-matter density in the left prefrontal, parahippocampal and bilateral posterior cerebellar regions, which could be more consistent with the notion of a distributed semantic-control network supporting the flexible retrieval and selection of word meaning.^52^ Interestingly, the involvement of the precentral cortex extends this pattern beyond canonical semantic regions, in line with recent evidence.^53^ Taken together, these findings do not align well with a disintegration of multimodal concepts occurring in a semantic ‘hub’,^54,55^ but rather point to an access semantic disorder in bvFTD, reflecting preserved semantic representations but impaired access to meaning, which reinforce the view of semantic cognition as a complex and distributed interactive system between control and representations dimensions.^52,56^

In AD, lexical impairments were substantial yet less pronounced than in bvFTD, with a particularly reduced performance in naming, as well as in fluency, relative to controls. Qualitative analyses showed higher semantic intrusions and repetitions (likely reflecting memory deficits) alongside with mild reduction in clustering and switching in fluency. In the word-comprehension tasks, patients showed intact access to target meanings and efficient rejection of close or distant distractors. This pattern suggests a functionally preserved semantic representational system, where retrieval inefficiency stems primarily from executive and memory-related constraints. At the neural level, lexical performance correlated with grey-matter reductions in bilateral frontal (superior and medial) and temporal regions, including the temporal poles and posterior inferior temporal gyri. Frontal involvement likely reflects increased executive and strategic demands during word retrieval, whereas temporal correlations could indicate inefficient access to lexical-semantic representations. Together, these findings point to a distributed frontotemporal dysfunction underlying lexical deficit in AD, reflecting access and mnemonic impairments. These results suggest that semantic access impairments are the first sign of the expected progressive semantic weakening over disease course.^56,57^

In PPD, lexical performance was moderately reduced relative to controls, with fewer words produced, diminished clustering and switching in fluency tasks, alongside naming difficulties reflecting mild word-finding difficulties. Error monitoring was preserved, as semantic intrusions and repetitions were not increased. Comprehension was also somewhat slightly weakened, with a few incorrect judgments regarding semantically related distractors. This pattern also suggests a mild inefficiency of executive-semantic control mechanisms rather than a degradation of conceptual knowledge. The absence of focal atrophy correlates further supports a diffuse, non-specific dysexecutive contribution to lexical and semantic regulation. This aligns with prior findings showing mild and variable fluency deficit in MDD, influenced by age and education^58^ and more consistent, moderate impairments in BD,^59^ with limited evidence for naming difficulties.

At the syntactic level, between-group differences were modest. Patients with bvFTD showed consistent impairments, particularly in sentence production, whereas only partial or mild difficulties were observed in AD and PPD in comparison with controls. These findings align with previous descriptions of reduced syntactic complexity and morphosyntactic omissions in bvFTD.^22^ The performance of AD and PPD groups suggests an overall preserved syntactic processing. This also aligns with observations of sentence-level difficulties in moderate-to-severe AD and schizophrenia likely reflecting increased memory or attentional demands rather than core syntactic deficits.^60-62^ Regarding neuroimaging correlations, no significant associations were observed in bvFTD or PPD. In AD, temporal and superior-frontal regions were involved, but these effects disappeared when controlling for the lexical composite score, underscoring the dependence of syntactic processing on lexico-semantic scaffolding.

Discourse measures yielded the largest between-group differences. Patients with bvFTD displayed pervasive impairments across fluency, informativity and coherence, with marked pragmatic violations. AD and PPD showed milder decrements, primarily reflecting reduced integrative capacity^63^ and executive load rather than pragmatic disorganization. In PPD, written-text comprehension was also reduced, possibly reflecting nonspecific cognitive demands rather than a genuine comprehension loss. Overall, discourse performance remained largely preserved in PPD, with subtle alterations consistent with the lexical inefficiencies described above, apart from a mild prosodic disturbance. These subtle pragmatic deviations align with previous reports in psychiatric disorders.^27,29,30,64^ Neuroanatomically, discourse performance involved distributed frontal, temporal and parietal regions across groups, predominantly left. In bvFTD, correlates disappeared after controlling for lower-level scores, suggesting that discursive breakdowns may be largely driven by impairments in linguistic access, control, and monitoring that are already evident at earlier processing levels.

Tasks involving cross-modal conversion (e.g. oral ↔ written) revealed additional but limited impairments, again mostly in bvFTD. Word and non-word repetition, as well as word reading, were largely preserved, with only non-word repetition and reading altered in bvFTD and AD, respectively. Sentence repetition was impaired in bvFTD and PPD. Writing words under dictation was impaired across all groups, whereas sentence writing was lower in bvFTD and PPD and automatic writing only showed a subtle decrease in bvFTD. Overall, these results indicate that core phonological, orthographic and cross-modal conversion mechanisms are largely preserved across groups. The observed impairments emerged when tasks imposed higher demands on monitoring, sequencing, or short-term memory, suggesting that deficits in these supporting systems rather than a primary disruption of phonological or orthographic processes, account for the difficulties observed. *Post hoc* analyses showed that writing deficits in bvFTD systematically co-occurred with lexical impairments, arguing against an isolated cross-modal (e.g. grapheme-phoneme conversion) deficit. Neuroanatomically, bvFTD showed broad correlations across bilateral frontotemporal, insular, parietal, occipital and cerebellar regions (left-dominant), while AD involved bilateral frontal and anterior temporal regions and PPD only the precuneus. After controlling for lexical performance, no correlations were observed anymore, except in bvFTD, where correlations narrowed to left lateral temporo-parietal, insular and ventral temporo-occipital clusters.

In this study, we organized our assessment and analyses to follow a hierarchical organization of language processing.^2,20^ Our findings were consistent with the assumed architecture, with cascading relationships across linguistic levels. Although our study was not designed to formally test causal dependencies between levels, this pattern reflects a mechanistic link whereby deficits in lexical level propagate to syntactic construction and subsequently disrupt discourse coherence and pragmatics. Future work should clarify these interdependencies through mediation or structural modelling approaches. Beyond this hierarchical organization, we also considered a transposition/transcoding dimension, which offered a complementary perspective by targeting the cross-modal conversion mechanisms that enable language to operate flexibly across oral and written forms. Examining this component that was rarely investigated before^22^ therefore refines the hierarchical model by capturing aspects of linguistic flexibility and sensorimotor conversion that are not encompassed by classical lexical, syntactic, or discursive measures, and may prove particularly sensitive to early or subtle network disruptions in bvFTD, especially considering its broad neural correlates.

Beyond the question of how linguistic levels are neurocognitively organized lies the broader issue of how language relates to social cognition. Recent theoretical accounts have approached this relationship from different angles. Some models describe the language system as a specialized mechanism for encoding and transmitting information, largely separable from the neural machinery supporting thought and (social) cognition.^4^ Other perspectives emphasize that language use in natural interaction is intrinsically shaped by social-cognitive operations such as partner modelling, turn-taking prediction and interpersonal coordination.^5^ Whether social cognition merely modulates a core linguistic system or is constitutive of how language is implemented in humans therefore remains an open question. Our study contributes to this debate by characterizing language functioning in bvFTD, a prototypical model of social-cognitive deficits.^12,13,33,65^ We observed robust discursive-level impairments in bvFTD. At this level, patients were required to speak to someone, thus engaging processes such as respecting turns, aligning with a partner’s lexical and informational choices, and monitoring interlocutor feedback. These alterations suggest genuine pragmatic and interactional difficulties that could be related to, or driven by, social cognitive deficits. However, the presence of lexical and syntactic alterations, together with access, control and monitoring impairment across all linguistic levels, indicate that discursive deficits in bvFTD more likely arise from multilayered disruption rather than a purely pragmatic mechanism. Future studies should explicitly disentangle the relative contribution of cognitive (including linguistic) determinants to pragmatic impairments in bvFTD. This cross-domain vulnerability is not unique to bvFTD; individuals with PPA also show alterations in social-cognitive functioning.^66^ Therefore, although it may be tempting to contrast bvFTD with primary progressive aphasia based on a putative dissociation between impaired social use of language versus disrupted core linguistic mechanisms, our findings instead support a dimensional view of FTD syndromes.^21^ Taken together, these patterns suggest that language and social cognition cannot be understood as strictly separable domains. Instead, they interact through a graded interface in which social-cognitive processes shape how linguistic mechanisms are deployed in real communication, and linguistic integrity constrains the expression of socially guided behaviour. bvFTD and PPA occupy different positions along this interface, but neither syndrome shows a ‘pure’ linguistic or ‘pure’ social-cognitive profile,^21^ with the right temporal predominant or semantic/behavioural variant of FTD being in between within this assumed spectrum.^66^ This supports a dimensional view in which communicative performance emerges from the continuous integration of partly distinct but interdependent systems.^21,67^

Clinically, our study shows that tasks assessing the lexical level are sensitive across diseases but lacked specificity, whereas discursive/pragmatic measures showed the greatest discriminative value for neurodegenerative diseases as compared to PPD and controls. In addition, our results indicate that language assessment does yield clinically actionable information for differentiating patients with bvFTD from other participants, as 85.5% of diagnostic accuracy was achieved through a handful of tasks, with moderate (57.6%) sensitivity but excellent (95.6%) specificity. These findings underline the value of speech analysis as a diagnostic marker of bvFTD and a potential outcome measure in clinical trials, because discourse integrates wider linguistic and socio-cognitive dimensions and may better reflect real-life communicative challenges. Future studies should leverage more automated, objective analyses of discourse and spontaneous speech in bvFTD such as through natural language processing pipelines, especially as recent findings indicate that they may good candidate to predict neuropathology^68,69^ and trajectories.^70,71^ By contrast, our study shows that qualitative analyses of responses during fluency tasks, which were consensually considered as promising,^44,72^ lack clinical discriminative power in a differential diagnosis context, especially when the total word count is taken into consideration in the interpretation.

Several limitations of the present study should be acknowledged. First, the PPD group was modest in size and diagnostically heterogeneous, comprising patients with MDD, BD and schizoaffective disorders, limiting disorder-specific profiling. Nevertheless, consistent with prior mixed-sample studies,^13,37,41-43,73^ clear language patterns emerged, underscoring the sensitivity of the multi-level framework. Future studies should include larger, diagnostically stratified cohorts, ideally with longitudinal assessments, to refine disorder-specific linguistic signatures. In addition, because patients with PPD were seen in a memory clinic in the context of a suspicion of bvFTD, we must emphasize that they do not present with typical psychiatric disorders, given their behavioural and/or cognitive symptoms that prompted referral for expert neurological evaluation. This was precisely our intention and, in our view, one of the main features that distinguishes our study from the existing literature, as we assess language abilities in patients in the most challenging clinical context, namely, when bvFTD is suspected. Second, all assessments were conducted in French, which may limit cross-linguistic generalization. French remains however underrepresented in research on language functioning in neurocognitive disorders and our study therefore contributes to diversifying the linguistic and cultural bases of the field.^^74-77^^ Cross-linguistic generalization is further shaped by structural differences across languages: in transparent orthographies (e.g. Finnish, Italian), grapheme–phoneme mappings may engage transcoding mechanisms differently from more opaque systems (e.g. English, Mandarin). Likewise, language with flexible word order and frequent embedded clauses (such as German) may impose greater syntactic demands. These variations highlight the need for cross-linguistic validation of multi-level language frameworks, especially to develop internationally standardized protocols^^78^^. Such diversification is essential to avoid Anglocentric bias and to ensure that clinical tools are valid across linguistic communities and diverse populations, and future studies should consider sociological modulators such as education, migration history, or multilingualism, which our study did not account for.^22^ A strength, however, is that our battery was rigorously controlled for psycholinguistic variables, such as frequency, length, concreteness and familiarity, ensuring internal validity.

In conclusion, this study showed that bvFTD presents multi-level language impairment with prominent discursive/pragmatic deficits and measurable transposition/transcoding costs, while AD and PPD exhibit broader lexical compromise with comparatively preserved syntax and milder discourse changes. Using a unified neurolinguistic framework, we showed that deficits at higher, more elaborated linguistic levels were not fully independent of deficits at lower levels. Convergent neuroanatomical associations support a distributed network organization that differentially recruits frontal–insular control systems and temporal semantic hubs across diseases. Embedding this framework into clinical workflows can improve diagnostic precision, provide accessible behavioural markers and facilitate translational endpoints for ecologically valid communicative outcomes.

## Supplementary Material

fcag116_Supplementary_Data
