# Supplementary material for: Multi-level language deficits in behavioural frontotemporal degeneration and related disorders
Source: Brain Commun. 2026 Apr 1;8(2):fcag116. doi: 10.1093/braincomms/fcag116 (PMC13075989; doi:10.1093/braincomms/fcag116)
Supplement: fcag116_Supplementary_Data [file fcag116_supplementary_data.docx]

**Multi-level language deficits in behavioural frontotemporal degeneration and related disorders**

**Supplementary Material**

1. **Supplementary Methods**

**1.1. Participants**

An initial group of 503 patients referred with suspected bvFTD was considered; 319 were excluded for the following reasons: a diagnosis of Lewy-Body disease (N = 4), Creutzfeldt-Jacob disease (N = 1), normal-pressure hydrocephalus (N = 7), encephalopathy (N = 16, due to anoxia, autoimmune disease, severe alcohol use disorder...), another FTLD diagnosis (N = 26, including progressive supranuclear palsy, corticobasal disease and primary progressive aphasia or predominant right temporal lobe FTD), vascular cognitive impairment (N = 43, including cerebral amyloid angiopathy), atypical Alzheimer’s disease (N = 48); presence of motor symptoms (N = 6, e.g. FTD with Amyotrophic Lateral Sclerosis); comorbidities likely impacting cognitive or affective processing (N = 8, e.g. intracranial tumor, sleep apnea syndrome, traumatic brain injury, epilepsy); diagnosis of possible bvFTD (N = 4); no diagnosis due to insufficient follow-up or subtle to mild cognitive impairment without clear progression (N = 156).

During this process, 50 patients fulfilling a DSM-5 psychiatric disorder were excluded due to significant comorbidities (e.g., significant leukopathy with Fazekas score > 1, history of stroke with sequelae, substance use disorders, severe alcohol use disorder) that could confound diagnosis. The selection process resulted in 58 patients who fulfilled the criteria for probable bvFTD, and 46 patients who were diagnosed with a psychiatric diagnosis, including MDD (59%), BD (35%) and schizoaffective disorder (6%) after bvFTD was excluded. Patients with BD were all assessed in the depressive phase, except for one patient who was in a manic phase at the time of testing. During this session, this patient exhibited partially inappropriate speech, was only partially cooperative, and demonstrated a tendency towards effort minimisation.

30 patients fulfilling McKhann et al. (2011) criteria for typical AD diagnosis and performing language assessment were included.

49 patients (including 24 with bvFTD and 25 with PPD) with insufficient language assessment were then excluded.

**1.2. Procedure**

**Multi-level qualitative language assessment**

**Lexical level > Fluency-based qualitative analyses**

Cluster and switch measures were collected following the procedures described by Troyer and colleagues¹ and their adaptation by Ledoux and colleagues², with adjustments for the present study. Because the semantic and phonemic subcategories defined in those previous studies were based on *animal* fluency and "F”, “A”, “S” letters task including, we adapted these subcategories in advance to correspond to *fruit* and “*V”*. The scoring rules from Troyer *et al.* (1997) and Ledoux *et al.* (2014) were adapted to the French language, and specifically to the *fruit* category, similarly to the adaptation performed for the Italian language³. They are summarized in **Supplementary Table 1**, along with illustrative (non-exhaustive) examples. Clusters and switches were calculated for both fluency tasks. A *cluster* was defined as a group of successively produced words belonging to the same subcategory, whereas a *switch* corresponded to a transition between clusters, including single, non-clustered words. Three cluster measures were computed: total cluster size, number of clusters, and mean cluster size.

*Total cluster size:* The sum of all clustered words. A cluster of size 1 corresponds to two successive words, a size of 2 corresponds to three successive words, and so on. For example, if a participant produces one cluster of two words and one cluster of three words, the total cluster size equals 3.

*Number of clusters:* The total number of multiword strings, each consisting of at least two successive words. Multiword strings are defined as sequences of two or more successive words whose relationship is determined by one of the adapted scoring rules.

*Mean cluster size:* The total cluster size, divided by the number of clusters.

Other specifications described in Troyer et al.^1^ and Ledoux et al.^2^ were also followed. Words identified as repetitions or intrusions were included within clusters. Words belonging to two successive clusters were counted twice, once as a member of each cluster. For example, in the sequence “*nectarine, apricot, cherry, strawberry, raspberry”*, the word *cherry* was counted both in the *stone fruits* cluster (*nectarine, apricot, cherry)* and in the *red fruits/berries* cluster (*cherry, strawberry, raspberry*). However, when a smaller cluster was entirely embedded within a larger one, only the larger cluster was scored. For example, all the words in the sequence “*vivre,* *vite, ville, vis, vice”* formed one cluster based on the shared initial letters (sharing the same first two letters), even though a subset (*vis, vice)* also met the criterion for homophony. In this case, only the cluster defined by the shared initial letters was scored. All scores were calculated by a single trained rater.

**Supplementary Table 1: Subcategories for clusters of semantic and phonemic fluency**

| **Subcategories (in French)** | **Examples (in French)** |
| --- | --- |
| **Semantic fluency “fruits”** | |
| Fruits à coque (*Nuts)* | Noix, noisette, amande, châtaigne, marron, noix de cajou, noix de macadamia, noix de pécan, pistache, noix de coco, faîne, gland… |
| Fruits exotiques (*Exotic fruits)* | Ananas, banane, kaki, litchi, kiwi, papaye, mangue, fruit de la passion, kumquat, fruit du dragon, avocat, goyave, grenade, noix de coco, datte, carambole, cacao, figue, mangoustan, physalis… |
| Fruits à pépins (*Pome fruits)* | Figue, coing, melon, pastèque, poire, pomme, kiwi, kaki, nashi, raisin, orange, clémentines, citron, mandarines, pamplemousse… |
| Agrumes (*Citrus fruits)* | Citron, cédrat, citron vert, clémentine, mandarine, orange, bigarade, calamondin, chinotto, combava, kumquat, orange sanguine, pamplemousse, pomelo, yuzu… |
| Fruits à noyau (*Stone fruits)* | Abricot, avocat, cerise, datte, griotte, litchi, mangue, brugnon, pêche, olive, nèfle du japon, prunes, pruneau, quetsche, mirabelle… |
| Fruits rouges/baies (*Red fruits/berries)* | Airelles, bleuet, canneberge, cassis, fraise, framboise, mûre, cerise, prunelle, groseille, myrtille, grenade, merise, alise, aveline… |
| **Phonological fluency “V”** | |
| Words with the same first two letters | Vache, valise, vasque (*Cow, suitcase, basin)* |
| Words with the same first and last sounds, differing only by a vowel sound (regardless of spelling) | Vie, vue, vent (*Life, sight, wind)* |
| Words that rhyme | Verrue, vertu (*Wart, virtue)* |
| Words that are homophones  (only counted if it is explicitly spelled by the participant) | Verre, vert, ver (*Glass, green, worm)* |

Examples for semantic subcategories have been taken from control participants’ answers.

*Lexical frequency.* Lexical frequency was computed using Lexique 3.83, a French corpus of approximately 140.000 words.^4^ For each word produced, frequency values were extracted based on subtitles’ occurrences, as these better reflect spoken language compared with frequency values extracted from novels.^5^ We used the *freqlemfilms* variable, representing lemma frequency per million occurrences in the subtitle’s corpus. This value corresponds to the sum of frequencies of all inflected forms of a given lemma (e.g., freq (tree) = freq (“tree”) + freq (“trees”)). When a word could correspond to multiple grammatical categories (e.g., travel as noun or verb), the higher frequency value was retained. Words absent from the corpus were assigned no frequency. For both semantic and phonemic fluency tasks, we computed the total lexical frequency for each participant, as well as four frequency subscores, following the methodology described by Lubineau et al.^6^: very frequent (greater than 100 per million), frequent (40 to 100 per million), rare (10 to 40 per million), and very rare (0 to 10 per million).

**Discursive level > Anamnestic interview**

The primary objective of this task was to collect the standard elements of clinical history as well as basic personal information (anamnesis). A secondary aim of this interview was to gather contextual information about the patients for an appropriate interpretation of their performance. This includes their premorbid language level, habitual use and management of spoken and written language, and any self-reported difficulties or complaints. Questions involved demographic information, professional status, medical and paramedical follow-up, home assistance, history of language disorder, patient’s self-reported difficulties, as well as hobbies, family status, children and grand-children, etc. Samples of spontaneous speech were also recorded afterwards for subsequent analysis. An analysis grid allowed to perform a quantitative and qualitative rating of participants’ fluency, lexical access, syntactic organisation, informativeness, comprehension, prosody, intelligibility, pragmatic aspects, and attention control.

**Discursive level > Narrative speech task**

The narrative speech task consisted of narrating a story composed of five sequential colour images presented in a predetermined order. The examiner positioned a screen between themselves and the participant so that only the participant could view the pictures. Three envelopes were then presented, each containing the same picture story, so that the participant believed they were making a choice unknown to the examiner. The participant is instructed: “Please choose one envelope. Look at the picture story inside and describe what is happening.” If the participant has difficulty understanding the instructions, they may be rephrased as: “Describe what is happening in this story. I will not look.” or “What is happening in these pictures?” At the end of the oral narrative, the participant was asked to imagine and provide the conclusion of the story. The analysis grid enabled both quantitative and qualitative screening of the patient’s discourse abilities, including lexicon, syntactic, informativity, pragmatic, actions and narrative quality were rated.

**Neuroimaging acquisition**

Whole-brain MRI scans were all acquired with eight channels head coil, obtained using either a 3T or a 1.5T scanner from different sites from the Hauts-de-France region. The different magnetic fields were well distributed in our groups (N = 10/22 MRI at 1,5T in the bvFTD group; N = 6/12 MRI at 1,5T in the PPD group and N = 7/15 at 1,5T in the AD group). MRI scanners used solely in one or two groups were excluded to prevent scanner-specific biases. In addition, only imaging examinations conducted within 12 months of the language assessment were considered. This resulted in 22 patients with bvFTD, 12 patients with PPD and 15 patients with AD with MRIs.

**Neuroimaging pre-processing**

MRI data were pre-processed using FSL-VBM^7,8^, part of the FSL software package.^9^ Structural images were brain-extracted using the BET brain extraction tool, and tissue segmentation was conducted using the FAST automatic segmentation tool.^10^ Rigorous quality checks were performed at each stage, with manual corrections performed (e.g. neck clean up, with manual editing if needed, robust brain centre estimation, modulation of fractional intensity threshold, etc.). Grey matter partial volumes were aligned to the Montreal Neurological Institute standard space (MNI152) using the FNIRT non-linear registration approach^11,12^, using a B-spline representation of the registration warp field.^13^ We created a study-specific template in which patients with bvFTD and LOAPD were equally represented, as well as images from each scanner, and the native grey matter images were registered non-linearly. Registered partial volumes maps were modulated by dividing them by the Jacobian modulation of the warp field to correct for local expansion or contraction. The Jacobian modulation step did not include the affine part of the registration, which meant that the data were normalized for head size as a scaling effect.^14^ Modulated images were smoothed with an isotropic Gaussian kernel with a sigma of 4 mm (roughly corresponding to 8mm full-width at half-maximum (FWHM) smoothing).

1. **Supplementary Results**

**2.1 Multi-level quantitative language assessment (Detailed results)**

The results (main scores) of the 23 tasks composing the battery are presented in **Supplementary Table 2** and the following paragraphs.

**Supplementary Table 2: Groups’ performance (mean of percentages) at the tasks composing the lexical level**

|  | **Mean ± SD** | | | |
| --- | --- | --- | --- | --- |
| **Domains**, *tasks* | **bvFTD (*n*=34)** | **AD (*n*=30)** | **PPD (*n*=21)** | **Controls (*n*=40)** |
| **Lexical level** |  |  |  |  |
| *Naming* |  |  |  |  |
| Objects | 83.33^*^ ± 10.91 | 79.35^*^ ± 15.78 | 87.17^*^ ± 9.40 | 93.12 ± 5.70 |
| Actions | 69.88^✝*^ ± 19.58 | 79.44^*^ ± 16.87 | 84.13^*^ ± 11.18 | 88.82 ± 5.78 |
| Famous people | 45.45^#*^ ± 27.40 | 37.67^*^ ± 27.75 | 68.00^*^ ± 20.67 | 79.75 ± 18.88 |
| *Comprehension* |  |  |  |  |
| Oral | 79.81^*^ ± 17.23 | 85.37^*^ ± 12.07 | 89.42^*^ ± 10.38 | 95.56 ± 5.06 |
| Written | 76.39^*^ ± 13.53 | 80.34^*^ ± 11.14 | 80.25 ± 14.16 | 88.33 ± 12.25 |
| *Fluency* |  |  |  |  |
| Action | 24.59^✝*^ ± 18.94 | 42.64^*^ ± 19.89 | 32.16^*^ ± 21.33 | 58.40 ± 21.12 |
| Semantic | 29.50^#*^ ± 14.97 | 35.00^*^ ± 15.06 | 44.26^*^ ± 14.45 | 59.41 ± 14.46 |
| Phonemic | 25.22^✝*^ ± 20.22 | 41.83^*^ ± 19.62 | 37.63^*^ ± 16.46 | 57.66 ± 19.78 |
| **Syntactic level** |  |  |  |  |
| Command execution | 90.23^*^ ± 15.12 | 94.83^*^ ± 11.87 | 94.44^*^ ± 12.17 | 99.17 ± 3.68 |
| Sentences production | 77.98^✝^ ± 25.28 | 92.31^*^ ± 13.52 | 88.89 ± 18.08 | 87.50 ± 17.19 |
| Sentences comprehension | 70.16^✝*^ ± 16.95 | 84.63 ± 10.97 | 76.19^*^ ± 16.78 | 87.50 ± 10.16 |
| **Discursive level** |  |  |  |  |
| Anamnestic interview | 89.76^#✝*^ ± 10.88 | 95.67^*^ ± 5.25 | 97.05^*^ ± 3.77 | 99.20 ± 1.34 |
| Narrative speech | 75.96^#✝*^ ± 14.38 | 87.70 ± 11.38 | 87.46 ± 14.53 | 90.83 ± 8.60 |
| Written texts comprehension | 80.00 ± 32.28 | 85.56 ± 28.61 | 80.00^*^ ± 29.42 | 91.67 ± 16.45 |
| **Transposition and transcoding** |  |  |  |  |
| *Repetition* |  |  |  |  |
| Words | 91.56 ± 13.70 | 96.21 ± 6.22 | 94.76 ± 8.73 | 94.75 ± 7.16 |
| Non-words | 97.37 ± 8.36 | 98.15^*^ ± 7.86 | 100.00 ± 0.00 | 91.67 ± 10.94 |
| Sentences | 65.91^*^ ± 25.63 | 74.17 ± 25.83 | 65.48^*^ ± 25.59 | 84.37 ± 20.94 |
| *Reading aloud* |  |  |  |  |
| Words | 97.47 ± 3.44 | 98.33 ± 2.59 | 97.14 ± 4.38 | 96.83 ± 3.37 |
| Non-words | 90.32^*^ ± 14.79 | 95.86 ± 6.02 | 94.00 ± 10.12 | 96.83 ± 5.00 |
| *Writing* |  |  |  |  |
| Words | 75.26^*^ ± 23.52 | 82.78^*^ ± 13.83 | 77.50^*^ ± 20.78 | 92.71 ± 8.27 |
| Non-words | 92.06 ± 17.17 | 92.06 ± 15.47 | 92.98 ± 13.96 | 90.00 ± 11.20 |
| Sentences | 86.83^*^ ± 12.93 | 90.74 ± 8.30 | 80.90^*^ ± 13.31 | 90.09 ± 7.47 |
| Automatic | 93.75^*^ ± 14.51 | 97.78 ± 7.24 | 97.50 ± 8.16 | 99.17 ± 3.68 |

Abbreviations*:* SD = standard deviation; bvFTD = behavioral variant frontotemporal degeneration; PPD = primary psychiatric disorder; AD = Alzheimer’s disease; *n* = sample size; ^*^vs controls (p < .05), ^#^vs PPD (p < .05), ^✝^vs AD (p < .05).

**Lexical level**

***Action fluency***

The bvFTD group showed significantly lower total score on the **action fluency task** compared with both the AD (*F=*12.755, *p<*.001; *ηp²=*.18; [–28.588, –8.052]) and control groups (*F=*45.982, *p<*.001; *ηp²=*.40; [–43.212, –23.562]). Similarly, the AD (*F=*7.861, *p=*.007; *ηp²=*.11; [–24.548, –4.128]) and PPD (*F=*23.697, *p<*.001; *ηp²=*.29; [–36.714, –15.313]) groups also performed worse than controls. No significant differences were observed between the bvFTD and PPD groups (*F=*1.990, *p=*.165; *ηp²=*.04; [–22.509, 3.942]), or between the AD and PPD groups (*F=*1.764, *p=*.191; *ηp²=*.04; [–5.017, 24.509]).

***Semantic fluency***

The bvFTD group showed significantly lower score on the **semantic fluency task** compared with both the PPD (*F=*9.603, *p=*.003; *ηp²=*.16; [–24.432, –5.216]) and control groups (*F=*67.269, *p<*.001; *ηp²=*.49; [–35.751, –21.762]). Similarly, the AD (*F=*27.959, *p<*.001; *ηp²=*.30; [–27.249, –12.312]) and PPD (*F=*19.492, *p<*.001; *ηp²=*.26; [–24.005, –9.024]) groups also performed worse than controls. No significant differences were observed between the AD and bvFTD groups (*F=*2.897, *p=*.094; *ηp²=*.05; [–1.173, 14.524]) or between the AD and PPD groups (*F=*0.589, *p=*.447; *ηp²=*.01; [–15.578, 6.977]).

***Phonological fluency***

All clinical groups showed significantly lower score on the **phonological fluency task** compared to controls, including bvFTD (*F=*43.264, *p<*.001; *ηp²=*.38; [–41.647, –22.263]), AD (*F=*9.134, *p=*.004; *ηp²=*.12; [–25.178, –5.146]) and PPD groups (*F=*17.992, *p<*.001; *ηp²=*.24; [–28.814, –10.333]). Similarly, the bvFTD group performed worse than the AD group (*F=*11.920, *p=*.001; *ηp²=*.17; [–28.514, –7.589]). No significant differences were retrieved between the PPD and the bvFTD groups (*F=*2.411, *p=*.127; *ηp²=*.05; [–2.749, 21.480]), and AD (*F=*1.977, *p=*.166; *ηp²=*.04; [–22.806, 4.042]).

***Object naming***

All clinical groups demonstrated a lower score on **object naming** compared to controls, including bvFTD (*F=*22.938, *p<*.001; *ηp²=*.25; [–13.758, –5.667]), AD (*F=*15.190, *p<*.001; *ηp²=*.19; [–16.872, –5.441]) and PPD groups (*F=*11.813, *p=*.001; *ηp²=*.17; [–10.018, –2.642]). No significant differences were observed between clinical groups (bvFTD vs PPD: *F=*2.825, *p=*.099; *ηp²=*.05; [–12.024, 1.069], bvFTD vs AD: *F=*0.863, *p=*.357; *ηp²=*.01; [–3.753, 10.261], AD vs PPD: *F=*0.644, *p=*.426; *ηp²=*.01; [–14.039, 6.031]).

***Action naming***

The bvFTD group demonstrated a lower score on **action naming** compared to the AD group (*F=*7.963, *p=*.007; *ηp²=*.12; [–21.827, –3.711]). Similarly, all clinical groups obtained a lower score compared to controls, including bvFTD (*F=*26.866, *p<*.001; *ηp²=*.28; [–22.825, –10.136]), AD (*F=*3.998, *p=*.050; *ηp²=*.06; [–12.482, –0.009]) and PPD groups (*F=*6.149, *p=*.016; *ηp²=*.10; [–9.798, –1.043]). No significant differences were obtained between the bvFTD (*F=*2.258, *p=*.139; *ηp²=*.04; [–18.332; 2.647]) and AD groups (*F=*0.009, *p=*.924; *ηp²=*.00; [–11.853, 10.772]) relative to PPD groups.

***Confrontational famous people naming***

The bvFTD group demonstrated a lower score on **famous people naming** compared to the PPD (*F=*6.900, *p=*.011; *ηp²=*.12; [–37.731, –5.023]) and control groups (*F=*40.258, *p<*.001; *ηp²=*.37; [–46.776, –24.398]). Similarly, the AD (*F=*41.608, *p<*.001; *ηp²=*39; [–51.529, –27.170]) and PPD groups (*F=*3.997, *p=*.050; *ηp²=*.07; [–21.357, 0.022]) showed a lower score compared to controls. No significant differences were obtained for the bvFTD (*F=*0.429, *p=*.515; *ηp²=*.01; [–9.544, 18.824]) and PPD groups (*F=*1.971, *p=*.167; *ηp²=*.04; [–5.183, 29.088]) compared to the AD group.

***Oral comprehension***

All clinical groups demonstrated a lower score on **oral comprehension** compared to controls, including bvFTD (*F=*27.583, *p<*.001; *ηp²=*.29; [–21.737, –9.762]), AD (*F=*17.291, *p<*.001; *ηp²=*.21; [–14.806, –5.200]), and PPD groups (*F=*7.399, *p=*.009; *ηp²=*.11; [–9.656, –1.467]). No significant differences were observed between clinical groups (bvFTD vs PPD: *F=*3.277, *p=*.077; *ηp²=*.07; [–18.963, 1.000], bvFTD vs AD: *F=*2.283, *p=*.136; *ηp²=*.04; [–13.997, 1.960], AD vs PPD: *F=*0.085, *p=*.772; *ηp²=*.00; [–10.076, 7.526]).

***Written comprehension***

The bvFTD and AD groups exhibited a lower score on **written comprehension** compared to controls (bvFTD: *F=*13.067, *p<*.001; *ηp²=*.18; [–19.145, –5.505], AD: *F=*8.363, *p=*.005; *ηp²=*.12; [–16.357, –2.986]). No significant differences were observed between the PPD and control groups (*F=*2.747, *p=*.103; *ηp²=*.05; [–13.553, 1.286]), as well as between clinical groups (bvFTD vs PPD: *F=*0.179, *p=*.675; *ηp²=*.00; [–12.103, 7.918], bvFTD vs AD: *F=*2.344, *p=*.133; *ηp²=*.05; [–12.289, 1.671], AD vs PPD: *F=*0.122, *p=*.729; *ηp²=*.00; [–12.299, 8.679]).

**Syntactic level**

***Command execution***

All clinical groups demonstrated a lower score on **command execution** compared to controls, including bvFTD (*F=*10.464, *p=*.002; *ηp²=*.14; [–13.607, –3.219]), AD (*F=*5.044, *p=*.028; *ηp²=*.07; [–9.385, –0.550]) and PPD (*F=*5.536, *p=*.022; *ηp²=*.09; [–9.381, –0.755]). No significant differences were obtained between clinical groups (bvFTD vs PPD: *F=*0.548, *p=*.463; *ηp²=*.01; [–12.951, 5.986], bvFTD vs AD: *F=*1.125, *p=*.294; *ηp²=*.02; [–11.308, 3.484], AD vs PPD: *F=*0.203, *p=*.654; *ηp²=*.00; [–11.417, 7.238]).

***Sentence production***

The bvFTD group obtained a lower score on **sentence production** compared to the AD group (*F=*5.934, *p=*.018; *ηp²=*.11; [–25.942, –2.495]). Surprisingly, the AD group exhibited a higher score compared to controls (*F=*4.407, *p=*.040; *ηp²=*.07; [0.427, 17.448]). No significant differences were retrieved for the bvFTD group compared to the PPD (*F=*2.477, *p=*.123; *ηp²=*.06; [–29.873, 3.696]) and control groups (*F=*2.130, *p=*.149; *ηp²=*.03; [–18.644, 2.903]), as for the PPD group compared to the AD (*F=*0.888, *p=*.352; *ηp²=*.02; [–17.760, 6.463]) and control groups (*F=*0.082, *p=*.776; *ηp²=*.00; [–11.452, 8.593]).

***Sentence comprehension***

The bvFTD group demonstrated a lower score on **sentence comprehension** compared to the AD (*F=*16.008, *p<*.001; *ηp²=*.22; [–23.135, –7.698]) and control groups (*F=*24.277, *p<*.001; *ηp²=*.27; [–23.391, –9.903]), as did the PPD group compared to controls (*F=*9.856, *p=*.003; *ηp²=*.15; [–17.730, –3.920]). No significant differences were observed for the PPD group compared to the bvFTD (*F=*0.603, *p=*.441; *ηp²=*.01; [–6.846, 15.457]) and AD groups (*F=*2.416, *p=*.127; *ηp²=*.05; [–17.262, 2.218]), as for the AD group compared to controls (*F=*1.416, *p=*.238; *ηp²=*.02; [–8.794, 2.227]).

**Discursive level**

***Anamnestic interview***

Specifically, the bvFTD group demonstrated a lower score on the **anamnestic interview** compared to the PPD (*F=*7.466, *p=*.009; *ηp²=*.13; [–13.460, –2.055]), AD (*F=*6.246, *p=*.015; *ηp²=*.10; [–10.325, –1.434]) and control groups (*F=*28.539, *p<*.001; *ηp²=*.29; [–13.134, –5.992]), as did the AD and PPD groups compared to controls (AD: *F=*13.415, *p<*.001; *ηp²=*.17; [–5.481, –1.614]; PPD: *F=*8.904, *p=*.004; *ηp²=*.13; [–3.427, –0.675]). No significant difference was observed between the AD and PPD groups (*F=*0.088, *p=*.768; *ηp²=*.00; [–4.060, 3.018]).

***Narrative speech***

Similarly, the bvFTD group presented a lower score on **narrative speech** compared to the PPD (*F=*7.584, *p=*.008; *ηp²=*.13; [–21.932, –3.432]), AD (*F=*11.231, *p=*.001; *ηp²=*.16; [–18.567, –4.681]), and control groups (*F=*33.412, *p<*.001; *ηp²=*.33; [–21.447, –10.441]). No significant differences were observed between the AD and PPD groups (*F=*2.180, *p=*.147; *ηp²=*.04; [–2.390, 15.550]), as for both groups compared to controls (AD vs controls: *F=*0.991, *p=*.323; *ηp²=*.01; [–7.564, 2.532], PPD vs controls : *F=*1.455, *p=*.233; *ηp²=*.02; [–8.701, 2.159]).

***Written text understanding***

No significant differences between groups were retrieved on **written text understanding** (bvFTD vs PPD: *F=*0.040, *p=*.841; *ηp²=*.00; [–23.044, 18.857], bvFTD vs AD: *F=*0.289, *p=*.593; *ηp²=*.00; [–20.877, 12.044], bvFTD vs controls: *F=*3.533, *p=*.065; *ηp²=*.05; [–23.696, 0.715], AD vs PPD: *F=*0.163, *p=*.688; *ηp²=*.00; [–18.448, 27.710], and AD vs controls: *F=*0.328, *p=*.569; *ηp²=*.00; [–15.721, 8.716]). The only exception was the PPD group which exhibited a lower score on written text understanding compared to controls (*F=*5.878, *p=*.019; *ηp²=*.09; [–25.515, –2.428]).

**Transposing/transcoding dimension**

***Repetition***

No significant differences were observed on **words repetition** for all groups (bvFTD vs PPD: *F=*2.879, *p=*.096; *ηp²=*.05; [–14.394, 1.214], bvFTD vs AD: *F=*2.226, *p=*.141; *ηp²=*.04; [–10.025, 1.464], bvFTD vs controls: *F=*1.977, *p=*.164; *ηp²=*.03; [–8.917, 1.545], AD vs PPD: *F=*0.530, *p=*.470; *ηp²=*.01; [–3.566, 7.609], AD vs controls: *F=*2.067, *p=*.155; *ηp²=*.03; [–0.954, 5.859], and PPD vs controls: *F=*0.001, *p=*.971; *ηp²=*.00; [–4.205, 4.360]).

No significant differences were obtained on **non**-**words repetition** for all groups (bvFTD vs PPD: *F=*4.008, *p=*.058; *ηp²=*.16; [–16.880, 0.321], bvFTD vs AD: *F=*0.024, *p=*.879; *ηp²=*.00; [–5.088, 5.921], bvFTD vs controls: *F=*3.164, *p=*.081; *ηp²=*.05; [–0.727, 12.208], AD vs PPD: *F=*0.602, *p=*.447; *ηp²=*.03; [–13.913, 6.368], and PPD vs controls: *F=*1.728, *p=*.196; *ηp²=*.04; [–3.640, 17.243]). The only exception was the AD group which demonstrated a higher score on non-words repetition compared to controls (*F=*4.457, *p=*.039; *ηp²=*.08; [0.383, 14.840]). Given the small sample size of the PPD group (n = 6) for this particular subscore, these results should be interpreted with caution.

The bvFTD and PPD groups demonstrated a lower score on **sentences repetition** compared to controls (bvFTD: *F=*10.158, *p=*.002; *ηp²=*.13; [–28.957, –6.662], PPD: *F=*9.909, *p=*.003; *ηp²=*.15; [–32.768, –7.287]). No significant differences were retrieved for the bvFTD group compared to the PPD (*F=*0.127, *p=*.723; *ηp²=*.00; [–13.538, 19.380]) and AD groups (*F=*2.063, *p=*.156; *ηp²=*.03; [–23.015, 3.782]), as for the AD group compared to the PPD (*F=*1.551, *p=*.219; *ηp²=*.03; [–32.139, 7.563]) and control groups (*F=*1.933, *p=*.169; *ηp²=*.03; [–21.120, 3.781]).

***Reading aloud***

No significant differences were observed on **reading aloud** **words** between groups (bvFTD vs PPD: *F=*0.091, *p=*.765; *ηp²=*.00; [–2.116, 2.861], bvFTD vs AD: *F=*0.901, *p=*.346; *ηp²=*.02; [–2.377, 0.847], bvFTD vs controls: *F=*0.382, *p=*.538; *ηp²=*.01; [–1.143, 2.169], AD vs PPD: *F=*0.195, *p=*.660; *ηp²=*.00; [–2.062, 3.224], AD vs controls: *F=*1.413, *p=*.239; *ηp²=*.02; [–0.660, 2.602], and PPD vs controls: *F=*0.510, *p=*.478; *ηp²=*.01; [–1.260, 2.655]).

No significant differences were observed on **reading aloud non-words** (bvFTD vs PPD: *F=*2.321, *p=*.134; *ηp²=*.05; [–15.477, 2.137], bvFTD vs AD: *F=*2.372, *p=*.129; *ηp²=*.04; [–10.774, 1.408], AD vs PPD: *F=*0.430, *p=*.515; *ηp²=*.01; [–4.221, 8.298], AD vs controls: *F=*1.405, *p=*.240; *ηp²=*.02; [–4.706, 1.200], PPD vs controls: *F=*1.197, *p=*.279; *ηp²=*.02; [–6.313, 1.853]). The only exception was the bvFTD group which exhibited a lower score on reading aloud non-words compared to controls (*F=*8.843, *p=*.004; *ηp²=*.12; [–12.745, –2.507]).

***Writing***

All clinical groups demonstrated a lower score on **writing words** compared to controls, including bvFTD (*F=*22.941, *p<*.001; *ηp²=*.25; [–27.514, –11.331]), AD (*F=*16.504, *p<*.001; *ηp²=*.20; [–16.658, –5.680]), and PPD groups (*F=*14.830, *p<*.001; *ηp²=*.21; [–21.156, –6.678]). No significant differences were observed between clinical groups (bvFTD vs PPD: *F=*1.690, *p=*.200; *ηp²=*.03; [–23.725, 5.093], bvFTD vs AD: *F=*1.256, *p=*.267; *ηp²=*.02; [–15.722, 4.436], AD vs PPD: *F=*0.172, *p=*.680; *ηp²=*.00; [–9.827, 14.936]).

No significant differences were observed on **writing non-words** (bvFTD vs PPD: *F=*0.301, *p=*.587; *ηp²=*.01; [–16.011, 9.197], bvFTD vs AD: *F=*0.282, *p=*.598; *ηp²=*.01; [–8.013, 13.719], bvFTD vs controls: *F=*0.462, *p=*.500; *ηp²=*.01; [–4.902, 9.938], AD vs PPD: *F=*0.008, *p=*.928; *ηp²=*.00; [–14.023, 15.333], AD vs controls: *F=*0.705, *p=*.405; *ηp²=*.01; [–4.746, 11.596], and PPD vs controls: *F=*0.013, *p=*.909; *ηp²=*.00; [–6.629, 7.440]).

The bvFTD and PPD groups demonstrated a lower score on **writing sentences** compared to controls (bvFTD: *F=*4.006, *p=*.050; *ηp²=*.06; [–9.855, –0.008], PPD: *F=*10.616, *p=*.002; *ηp²=*.16; [–13.672, –3.259]), with no difference between them (*F=*0.008, *p=*.927; *ηp²=*.00; [–8.132, 8.906]). No significant differences were retrieved for the AD group compared to the bvFTD (*F=*0.807, *p=*.373; *ηp²=*.01; [–8.253, 3.146]), PPD (*F=*3.658, *p=*.062; *ηp²=*.07; [–0.407, 15.707]), and control groups (*F=*0.013, *p=*.910; *ηp²=*.00; [–4.312, 3.848]).

***Automatic writing***

The bvFTD group exhibited a lower score on automatic writing compared to controls (*F=*4.293, *p=*.042; *ηp²=*.06; [–10.115, –0.190]). No other significant difference was observed on **automatic writing** (bvFTD vs PPD: *F=*0.700, *p=*.407; *ηp²=*.01; [–11.820, 4.873], bvFTD vs AD: *F=*1.945, *p=*.168; *ηp²=*.03; [–10.474, 1.872], AD vs PPD: *F=*0.275, *p=*.602; *ηp²=*.01; [–4.337, 7.396], AD vs controls: *F=*0.422, *p=*.518; *ηp²=*.01; [–3.856, 1.963], and PPD vs controls: *F=*1.395, *p=*.243; *ηp²=*.02; [–5.092, 1.315]).

**2.2 Multi-level qualitative language assessment**

In the following paragraphs, we performed a qualitative analysis according to the categories of items (e.g. living vs non-living objects), the types of errors (e.g. semantically close distractors vs semantically unrelated distractors), the strategic organization of responses (e.g. clustering and switching), or type of responses provided (e.g. lexical frequency). Results for naming and comprehension tasks are presented in **Supplementary Table 3**.

**Lexical level**

***Object naming***

All clinical groups showed a lower score on **living objects** compared to controls, including bvFTD (*F=*20.450, *p*<.001; *ηp²=*.23; [–2.465, –0.956]), AD (*F=*12.430, *p*<.001; *ηp²=*.16; [–3.358, –0.930]) and PPD groups (*F=*12.135, *p*<.001; *ηp²=*.18; [–2.550, –0.688]). No significant differences were retrieved between clinical groups (bvFTD vs PPD: *F=*0.532, *p=*.469; *ηp²=*.01; [–1.825, 0.853], bvFTD vs AD: *F=*1.543, *p=*.219; *ηp²=*.02; [–0.520, 2.221], AD vs PPD: *F=*0.056, *p=*.814; *ηp²=*.00; [–2.499, 1.972]).

All clinical groups demonstrated a lower score on **non-living objects** compared to controls, including bvFTD (*F=*15.130, *p*<.001; *ηp²=*.18; [–2.840, –0.914]), AD (*F=*13.920, *p*<.001; *ηp²=*.17; [–3.047, –0.923]) and PPD groups (*F=*6.582, *p*=.013; *ηp²=*.11; [–1.596, –0.196]). No significant differences were retrieved between clinical groups (bvFTD vs PPD: *F=*3.068, *p=*.086; *ηp²=*.06; [–2.766, 0.190], bvFTD vs AD: *F=*0.204, *p=*.653; *ηp²=*.00; [–1.101, 1.743], AD vs PPD: *F=*1.346, *p=*.252; *ηp²=*.03; [–2.815, 0.756]).

***Famous people naming***

All clinical groups demonstrated a lower score for **famous women** compared to controls, including bvFTD (*F=*49.848, *p<*.001; *ηp²=*.42; [–3.056, –1.709]), AD (*F=*42.966, *p<*.001; *ηp²=*.40; [–3.059, –1.630]) and PPD groups (*F=*8.478, *p=*.005; *ηp²=*.13; [–1.663, –0.307]). The bvFTD group showed a lower score compared to the PPD group (*F=*5.760, *p=*.020; *ηp²=*.11; [–2.374, –0.210]). No significant differences were observed for the bvFTD (*F=*0.043, *p=*.837; *ηp²=*.00; [–0.804, 0.989]) and PPD groups (*F=*1.075, *p=*.305; *ηp²=*.02; [–0.581, 1.813]) compared to the AD group.

The bvFTD and AD groups demonstrated a lower score for **famous men** compared to controls (bvFTD: *F=*17.030, *p<*.001; *ηp²=*.20; [–1.745, –0.608], AD: *F=*22.800, *p<*.001; *ηp²=*.26; [–2.124, –0.871]). No significant differences were obtained for the PPD group compared to controls (*F=*2.469, *p=*.122; *ηp²=*.04; [–1.127, 0.136]), as for the bvFTD group relative to the PPD (*F=*1.166, *p=*.286; *ηp²=*.02; [–1.272, 0.383]) and AD groups (*F=*0.633, *p=*.430; *ηp²=*.01; [–0.396, 0.918]), as well for the PPD group compared to the AD group (*F=*0.451, *p=*.505; *ηp²=*.01; [–0.597, 1.195]).

The bvFTD group showed a lower score for **famous artists** compared to controls (*F=*24.414, *p<*.001; *ηp²=*.26; [–2.960, –1.257]), as did the AD group (*F=*18.921, *p<*.001; *ηp²=*.22; [–2.993, –1.109]). No significant differences were obtained for the PPD group compared to controls (*F=*2.462, *p=*.122; *ηp²=*.04; [–1.554, 0.189), as the bvFTD group compared to the PPD group (*F=*3.187, *p=*.080; *ηp²=*.06; [–2.487, 0.147]) and AD (*F=*0.002, *p=*.962; *ηp²=*.00; [–1.083, 1.136]), and the PPD group relative to the AD group (*F=*0.032, *p=*.858; *ηp²=*.00; [–1.316, 1.574]).

All clinical groups showed a lower score for **famous politicians** compared to controls, including bvFTD (*F=*42.599, *p<*.001; *ηp²=*.38; [–1.893, –1.007]), AD (*F=*66.832, *p<*.001; *ηp²=*.51; [–2.229, –1.354]) and PPD groups (*F=*10.008, *p=*.003; *ηp²=*.15; [–1.304, –0.293]). No significant differences were retrieved for the bvFTD group compared to the PPD (*F=*2.929, *p=*.093; *ηp²=*.06; [–1.232, 0.099]) and AD groups (*F=*1.840, *p=*.180; *ηp²=*.03; [–0.156, 0.810]). The AD group demonstrated a lower score compared to the PPD group (*F=*5.088, *p=*.029; *ηp²=*.10; [–1.487, –0.084]).

***Oral comprehension***

No significant differences were obtained on **correct target score** for all groups (bvFTD vs PPD: *F=*0.452, *p=*.505; *ηp²=*.01; [–0.587, 0.293], bvFTD vs AD: *F=*0.426, *p=*.517; *ηp²=*.01; [–0.456, 0.232], bvFTD vs controls: *F=*2.218, *p=*.141; *ηp²=*.03; [–0.504, 0.073], AD vs PPD: *F=*0.118, *p=*.732; *ηp²=*.00; [–0.371, 0.263], AD vs controls: *F=*2.125, *p=*.150; *ηp²=*.03; [–0.369, 0.058], PPD vs controls: *F=*0.178, *p=*.675; *ηp²=*.00; [–0.151, 0.232]).

The bvFTD group demonstrated a lower score on **semantically related errors** compared to the AD (*F=*5.096, *p=*.028; *ηp²=*.08; [–3.967, –0.238]) and control groups (*F=*14.697, *p<*.001; *ηp²=*.18; [–4.579, –1.444]). Similarly, the PPD group showed a lower score compared to controls (*F=*4.826, *p=*.032; *ηp²=*.08; [–1.404, –0.064]). No significant differences were retrieved for the bvFTD group compared to the PPD (*F=*3.452, *p=*.069; *ηp²=*.07; [–4.958, 0.197]) and AD groups (*F=*1.119, *p=*.296; *ηp²=*.02; [–1.801, 0.561]), as for the AD group compared to controls (*F=*3.838, *p=*.054; *ηp²=*.06; [–1.265, 0.012]), although a statistical trend could be observed.

No significant differences were observed for **semantically far errors** between all groups (bvFTD vs PPD: *F=*1.342, *p=*.252; *ηp²=*.03; [–3.786, 1.019], bvFTD vs AD: *F=*1.568, *p=*.216; *ηp²=*.03; [–2.805, 0.647], bvFTD vs controls: *F=*3.696, *p=*.059; *ηp²=*.05; [–2.885, 0.054], AD vs PPD: *F=*1.327, *p=*.256; *ηp²=*.03; [–0.233, 0.855], AD vs controls: *F=*0.354, *p=*.554; *ηp²=*.01; [–0.342, 0.185], PPD vs controls: *F=*3.513, *p=*.066; *ηp²=*.06; [–0.638, 0.021]).

No significant differences were obtained on **semantically unrelated errors** between all groups (bvFTD vs PPD: *F=*1.177, *p=*.284; *ηp²=*.02; [–3.657, 1.095], bvFTD vs AD: *F=*1.809, *p=*.184; *ηp²=*.03; [–2.860, 0.562], bvFTD vs controls: *F=*2.773, *p=*.100; *ηp²=*.04; [–2.677, 0.241], AD vs PPD: *F=*1.992, *p=*.165; *ηp²=*.04; [–0.269, 0.047], AD vs controls: *F=*0.443, *p=*.508; *ηp²=*.01; [–0.150, 0.075], PPD vs controls: *F=*0.132, *p=*.718; *ηp²=*.00; [–0.061, 0.087]).

***Written comprehension***

No significant differences were obtained on **correct target score** between all groups (bvFTD vs PPD: *F=*0.059, *p=*.810; *ηp²=*.00; [–0.592, 0.753], bvFTD vs AD: *F=*3.298, *p=*.076; *ηp²=*.07; [–0.806, 0.041], bvFTD vs controls: *F=*0.008, *p=*.930; *ηp²=*.00; [–0.527, 0.575], AD vs PPD: *F=*0.360, *p=*.552; *ηp²=*.01; [–0.781, 0.424], AD vs controls: *F=*2.209, *p=*.142; *ηp²=*.03; [–0.138, 0.939], PPD vs controls: *F=*0.307, *p=*.582; *ηp²=*.00; [–0.848, 0.481]).

No significant differences were obtained for **semantically related errors** between all groups (bvFTD vs PPD: *F=*0.003, *p=*.955; *ηp²=*.00; [–1.538, 1.627], bvFTD vs AD (*F=*0.327, *p=*.570; *ηp²=*.01; [–1.432, 0.799], bvFTD vs controls: *F=*0.967, *p=*.329, *ηp²=*.02; [–1.382, 0.471], AD vs PPD: *F=*0.003, *p=*.956; *ηp²=*.00; [–1.856, 1.961], AD vs controls: *F=*0.362, *p=*.549; *ηp²=*.01; [–1.415, 0.760], PPD vs controls: *F=*0.021, *p=*.885; *ηp²=*.00; [–1.291, 1.116]).

No significant differences were obtained for **semantically far errors** between all groups (bvFTD vs PPD: *F=*1.750, *p=*.194; *ηp²=*.05; [–0.735, 0.155], bvFTD vs AD: *F=*3.906, *p=*.054; *ηp²=*.08; [–0.749, 0.007], AD vs PPD: *F=*0.129, *p=*.722; *ηp²=*.00; [–0.394, 0.564], AD vs controls: *F=*0.664, *p=*.418; *ηp²=*.01; [–0.394, 0.166], PPD vs controls: *F=*3.513, *p=*.066; *ηp²=*.06; [–0.638, 0.021]). The only exception was the bvFTD group which exhibited significantly lower score on semantically far compared to controls (*F=*10.471, *p=*.002; *ηp²=*.15; [–0.719, –0.170]).

No significant differences were obtained for **semantically unrelated errors** between all groups (bvFTD vs AD: *F=*1.032, *p=*.315; *ηp²=*.02; [–0.168, 0.055], bvFTD vs controls: *F=*1.203, *p=*.277; *ηp²=*.02; [–0.198, 0.058], AD vs PPD: as both performances were equal to 100%, No significant differences could be observed, AD vs controls: *F=*0.197, *p=*.659; *ηp²=*.00; [–0.076, 0.119], PPD vs controls: *F=*1.407, *p=*.241; *ηp²=*.03; [–0.047, 0.182]). The only exception was the bvFTD group which demonstrated significantly lower score on semantically unrelated compared to the PPD group (*F=*4.548, *p=*.040; *ηp²=*.11; [–0.328, –0.008]).

**Supplementary Table 3: Quantitative indicators in the Famous people naming task and the oral and written comprehension tasks in patients and control groups**

|  | **Mean ± SD** | | | |
| --- | --- | --- | --- | --- |
| **Tasks**, *indicators* | **bvFTD** | **AD** | **PPD** | **Controls** |
| **Object naming** | | | | |
| *N* | 33 | 30 | 20 | 40 |
| Living (/18) | 14.85^*^ ± 1.84 | 13.83^*^ ± 3.25 | 15.05^*^ ± 2.46 | 16.60 ± 1.32 |
| Non-living (/18) | 15.15^*^ ± 2.67 | 14.73^*^ ± 2.84 | 16.10^*^ ± 1.37 | 17.00 ± 1.18 |
| **Famous people naming** |  |  |  |  |
| *N* | 33 | 29 | 20 | 40 |
| Women (/4) | 2.55^#*^ ± 1.73 | 2.38^*^ ± 1.63 | 3.75^*^ ± 1.48 | 4.83 ± 1.03 |
| Men (/4) | 2.00^*^ ± 1.27 | 1.55^*^ ± 1.35 | 2.55 ± 1.32 | 3.15 ± 1.08 |
| Artists (/7) | 3.55^*^ ± 2.11 | 3.28^*^ ± 2.19 | 4.85 ± 1.84 | 5.60 ± 1.37 |
| Politicians (/3) | 1.00^*^ ± 1.00 | 0.65^*^ ± 0.81 | 1.45^✝*^ ± 1.05 | 2.38 ± 0.87 |
| **Oral comprehension** | | | | |
| *N* | 32 | 29 | 19 | 40 |
| Correct target (/18) | 17.66 ± 0.78 | 17.72 ± 0.45 | 17.89 ± 0.32 | 17.87 ± 0.33 |
| Sem. related error (/18) | 14.12^✝*^ ± 4.63 | 16.17 ± 1.54 | 16.37^*^ ± 1.61 | 17.12 ± 0.91 |
| Sem. far error (/18) | 16.53 ± 4.32 | 17.65 ± 0.61 | 17.63 ± 0.83 | 17.87 ± 0.40 |
| Sem. unrelated error (/18) | 16.78 ± 4.41 | 17.93 ± 0.26 | 18.0 ± 0.00 | 17.97 ± 0.16 |
| **Written comprehension** | | | | |
| *N* | 24 | 26 | 16 | 40 |
| Correct target (/18) | 17.33 ± 0.87 | 17.69 ± 0.55 | 17.31 ± 0.95 | 17.42 ± 1.15 |
| Sem. related error (/18) | 15.08 ± 1.86 | 15.06 ± 1.79 | 15.08 ± 2.42 | 15.60 ± 1.79 |
| Sem. far error (/18) | 17.37^*^ ± 0.71 | 17.65 ± 0.63 | 17.75 ± 0.45 | 17.82 ± 0.57 |
| Sem. unrelated error (/18) | 17.92^#^ ± 0.28 | 18.00 ± 0.00 | 18.00 ± 0.00 | 17.95 ± 0.24 |

Number of responses according to words’ category and types of errors. Abbreviations*:* SD = standard deviation; bvFTD = behavioral variant frontotemporal degeneration; PPD = primary psychiatric disorder; AD = Alzheimer’s disease; *N* = sample size; % = percentage; Sem= semantic; ^*^vs controls (p < .05), ^#^vs PPD (p < .05), ^✝^vs AD (p < .05).

***Semantic fluency***

The bvFTD group produced significantly fewer **semantic words** than the AD (*F=*6.766, *p=*.012; *ηp²=*.11; [–6.327, –0.820]) and control groups (*F=*43.696, *p<*.001; *ηp²=*.41; [–11.150, –5.974]). Similarly, the AD (*F=*11.808, *p=*.001; *ηp²=*.16; [–7.439, –1.967]) and PPD groups (*F=*24.090, *p<*.001; *ηp²=*.32; [–9.670, –4.056]) also performed worse than controls. No significant differences were found between the bvFTD and PPD groups (*F=*0.914, *p=*.344; *ηp²=*.02; [–5.394, 1.923]), nor between the AD and PPD groups (*F=*1.765, *p=*.191; *ηp²=*.04; [–1.370, 6.661]).

With the exception of the AD group which produced significantly more semantic repetitions than controls (*F=*10.068, *p=*.002; *ηp²=*.14; [0.655, 2.883]), no significant group differences were observed regarding the number of **semantic repetitions** (bvFTD vs PPD: *F=*2.279, *p=*.138; *ηp²=*.05; [–0.338, 2.355], bvFTD vs AD: *F=*3.697, *p=*.060; *ηp²=*.06; [–2.535, 0.052], bvFTD vs controls: *F=*2.602, *p=*.112; *ηp²=*.04; [–0.166, 1.552], AD vs PPD: *F=*3.297, *p=*.076; *ηp²=*.07; [–0.188, 3.589], PPD vs controls: *F=*0.005, *p=*.945; *ηp²=*.00; [–0.772, 0.721]).

The bvFTD group produced significantly more **semantic intrusions** than controls (*F=*5.374, *p=*.024; *ηp²=*.08; [0.074, 0.997]), as did the AD group (*F=*5.727, *p=*.020; *ηp²=*.08; [0.062, 0.688]). No significant difference was found between the PPD and control groups (*F=*2.495, *p=*.120; *ηp²=*.05; [–0.075, 0.626]). Similarly, no significant differences were observed between clinical groups (bvFTD vs PPD: *F=*0.715, *p=*.402; *ηp²=*.02; [–0.489, 1.197], bvFTD vs AD: *F=*0.084, *p=*.773; *ηp²=*.00; [–0.470, 0.629], AD vs PPD: *F=*0.058, *p=*.810; *ηp²=*.00; [–0.564, 0.717]).

All clinical groups showed significantly a smaller **semantic total cluster size** compared with controls, including bvFTD (*F=*25.872, *p<*.001; *ηp²=*.29; [–5.857, –2.553]), AD (*F=*6.291, *p=*.015; *ηp²=*.09; [–4.194, –0.474]) and PPD (*F=*16.651, *p<*.001; *ηp²=*.25; [–6.217, –2.114]). No significant differences were observed between the clinical groups (bvFTD vs PPD: *F=*0.022, *p=*.884; *ηp²=*.00; [–2.576, 2.226], bvFTD vs AD: *F=*3.729, *p=*.059; *ηp²=*.06; [–3.375, 0.063], AD vs PPD: *F=*1.949, *p=*.170; *ηp²=*.04; [–0.896, 4.907]). Interestingly, no group differences remained after controlling for the total number of semantically evoked words.

All clinical groups showed significantly lower **semantic clusters number** compared with controls, including bvFTD (*F=*25.133, *p<*.001; *ηp²=*.29; [–3.267, –1.405]), AD (*F=*9.008, *p=*.004; *ηp²=*.13; [–2.434, –0.488]) and PPD (*F=*15.178, *p<*.001; *ηp²=*.24; [–2.989, –0.955]). No significant differences were observed between clinical groups (bvFTD vs PPD: *F=*0.002, *p=*.969; *ηp²=*.00; [–1.513, 1.456], bvFTD vs AD: *F=*3.135, *p=*.082; *ηp²=*.05; [–2.017, 0.125], AD vs PPD: *F=*0.904, *p=*.347; *ηp²=*.02; [–0.860, 2.389]). Interestingly, no group differences remained after controlling for the number of semantically evoked words.

The PPD group showed significantly smaller **semantic mean cluster size** compared with both the AD (*F=*5.000, *p=*.031; *ηp²=*.11; [–0.866, –0.044]) and controls groups (*F=*6.433, *p=*.014; *ηp²=*.12; [–0.494, –0.057]). No significant differences were found for the bvFTD group relativelty to PPD (*F=*0.005, *p=*.944; *ηp²=*.00; [–0.334, 0.358]), AD (*F=*0.053, *p=*.819; *ηp²=*.00; [–0.325, 0.258]) and controls (*F=*1.358, *p=*.248; *ηp²=*.02; [–0.365, 0.096]), nor for the AD group compared with controls (*F=*0.010, *p=*.921; *ηp²=*.00; [–0.244, 0.269]). Interestingly, no group differences remained after controlling for the number of semantically evoked words.

The bvFTD group showed significantly lower **semantic switches number** compared with both the AD (*F=*6.904, *p=*.011; *ηp²=*.11; [–3.827, –0.515]) and controls groups (*F=*38.833, *p<*.001; *ηp²=*.38; [–5.484, –2.821]), as did the AD (*F=*6.680, *p=*.012; *ηp²=*.10; [–3.291, –0.420]) and PPD groups (*F=*17.883, *p<*.001; *ηp²=*.27; [–4.283, –1.524]) compared to controls. No significant differences were observed between the bvFTD and PPD groups (*F=*0.907, *p=*.346; *ηp²=*.02; [–3.245, 1.164]), nor between the AD and PPD groups (*F=*2.124, *p=*.153; *ηp²=*.05; [–0.687, 4.252]). Interestingly, no group differences remained after controlling for the number of semantically evoked words.

The total **frequency of words** produced was significantly lower for all clinical groups compared with controls, including bvFTD (*F=*15.116, *p<*.001; *ηp²=*.19; [–73.253, –23.516]), AD (*F=*6.137, *p=*.016; *ηp²=*.09; [–66.840, –7.141]), and PPD (*F=*5.437, *p=*.024; *ηp²=*.10; [–68.527, –5.118]). No significant differences were retrieved between the clinical groups (bvFTD vs PPD: *F=*0.132, *p=*.718; *ηp²=*.00; [–44.830, 31.132], bvFTD vs AD: *F=*0.521, *p=*.474; *ηp²=*.01; [–38.829, 18.266], AD vs PPD: *F=*0.134, *p=*.716; *ηp²=*.00; [–40.078, 57.830]). Interestingly, no group differences remained after controlling for the number of semantically correct words.

No significant differences between the groups were retrieved on the amount of **very frequent words** produced (bvFTD vs PPD: *F=*1.207, *p=*.278; *ηp²=*.03; [–0.320, 0.094], bvFTD vs AD: *F=*3.209, *p=*.079; *ηp²=*.06; [–0.306, 0.017], bvFTD vs controls: *F=*0.876, *p=*.353; *ηp²=*.01; [–0.202, 0.073], AD vs PPD: *F=*0.221, *p=*.641; *ηp²=*.00; [–0.245, 0.394], AD vs controls: *F=*0.945, *p=*.335; *ηp²=*.01; [–0.098, 0.283], and PPD vs controls: *F=*0.276, *p=*.602; *ηp²=*.00; [–0.153, 0.262]).

The PPD and AD groups produced a significantly higher number of **frequent words** compared with controls (PPD: *F=*4.616, *p=*.036; *ηp²=*.08; [–0.431, –0.015]; AD: *F=*4.314, *p=*.042; *ηp²=*.06; [–0.351, –0.007]). The bvFTD group generated more frequent words than the PPD group (*F=*4.790, *p=*.034; *ηp²=*.10; [0.033, 0.794]), but did not differ from controls (*F=*0.109, *p=*.742; *ηp²=*.00; [–0.186, 0.260]) or the AD group (*F=*1.135, *p=*.291; *ηp²=*.02; [–0.121, 0.395]). No significant differences were found between the AD and PPD groups (*F=*0.148, *p=*.703; *ηp²=*.00; [–0.269, 0.396]). Only the difference between the bvFTD and PPD groups remained significant after controlling the number of semantically correct words.

All clinical groups produced significantly fewer **rare words** compared to controls, including bvFTD (*F=*21.471, *p<*.001; *ηp²=*.25; [–2.666, –1.059]), AD (*F=*16.955, *p<*.001; *ηp²=*.22; [–2.529, –0.876]), and PPD groups (*F=*13.795, *p<*.001; *ηp²=*.21; [–2.666, –0.795]), with no significant differences observed between the clinical groups (bvFTD vs PPD: *F=*0.458, *p=*.502; *ηp²=*.01; [–1.387, 0.690], bvFTD vs AD: *F=*0.000, *p=*.985; *ηp²=*.00; [–0.770, 0.756], AD vs PPD: *F=*0.036, *p=*.850; *ηp²=*.00; [–1.012, 1.223]). Interestingly, no group differences remained after controlling for the number of semantically correct words.

All clinical groups produced fewer **very rare words** compared to controls, including bvFTD (*F=*42.716, *p<*.001; *ηp²=*.40; [–8.383, –4.457]), AD (*F=*20.621, *p<*.001; *ηp²=*.25; [–6.501, –2.527]), and PPD groups (*F=*13.166, *p<*.001; *ηp²=*.21; [–6.058, –1.742]), with No significant differences observed between the clinical groups (bvFTD vs PPD: *F=*3.886, *p=*.055; *ηp²=*.08; [–5.723, 0.063], bvFTD vs AD: *F=*1.919, *p=*.172; *ηp²=*.03; [–3.554, 0.649], and AD vs PPD: *F=*0.004, *p=*.949; *ηp²=*.00; [–2.949, 3.144]). Interestingly, no group differences remained after controlling for the number of semantically correct words.

***Phonological fluency***

The bvFTD group produced significantly fewer **phonological words** than the AD group (*F=*15.192, *p<*.001; *ηp²=*.21; [–10.420, –3.347]). Similarly, the bvFTD (*F=*35.982, *p<*.001; *ηp²=*.36; [–12.420, –6.216]) and PPD groups (*F=*17.286, *p<*.001; *ηp²=*.25; [–9.833, –3.429]) performed produced fewer words than controls. No significant differences were observed between the bvFTD and PPD groups (*F=*0.529, *p=*.471; *ηp²=*.01; [–5.635, 2.647]), as between the AD and PPD groups (*F=*3.207, *p=*.080; *ηp²=*.07; [–0.556, 9.379]) and between the AD and control groups (*F=*3.433, *p=*.069; *ηp²=*.05; [–6.554, 0.247]) groups.

Except for the AD group, which produced significantly more phonological repetitions compared to controls (*F=*6.417, *p=*.014; *ηp²=*.09; [0.202, 1.712]), no other significant group differences were observed regarding the number of **phonological repetitions** (bvFTD vs PPD: *F=*0.794, *p=*.378; *ηp²=*.02; [–0.287, 0.742], bvFTD vs AD: *F=*3.656, *p=*.061; *ηp²=*.06; [–1.458, 0.034], bvFTD vs controls: *F=*2.144, *p=*.148; *ηp²=*.03; [–0.102, 0.664], AD vs PPD: *F=*1.197, *p=*.280; *ηp²=*.03; [–0.567, 1.911], PPD vs controls: *F=*0.021, *p=*.885; *ηp²=*.00; [–0.456, 0.395]).

No significant group differences were obtained in the number of **phonological intrusions** (bvFTD vs PPD: *F=*0.515, *p=*.477; *ηp²=*.01; [–0.276, 0.582], bvFTD vs AD: *F=*0.437, *p=*.511; *ηp²=*.01; [–0.713, 0.359], bvFTD vs controls: *F=*0.592, *p=*.445; *ηp²=*.01; [–0.196, 0.442], AD vs PPD: *F=*0.282, *p=*.598; *ηp²=*.01; [–0.612, 1.050], AD vs controls: *F=*1.041, *p=*.312; *ηp²=*.02; [–0.259, 0.799], PPD vs controls: *F=*0.344, *p=*.560; *ηp²=*.01; [–0.424, 0.232]).

The bvFTD group showed a significantly smaller **phonological total cluster size** compared to the AD (*F=*6.419, *p=*.014; *ηp²=*.10; [–3.938, –0.461]) and control groups (*F=*13.315, *p<*.001; *ηp²=*.17; [–5.613, –1.642]), as did the PPD group relative to controls (*F=*8.173, *p=*.006; *ηp²=*.14; [–5.274, –0.921]).. No significant differences were found for the PPD relative to the bvFTD (*F=*0.078, *p=*.782; *ηp²=*.00; [–2.819, 2.135]) and AD groups (*F=*0.238, *p=*.628; *ηp²=*.01; [–2.819, 1.721]), nor between the AD and control groups (*F=*3.656, *p=*.060; *ηp²=*.05; [–3.719, 0.082]). Interestingly, no group differences remained after controlling for the number of phonologically words

The bvFTD group demonstrated significantly lower **phonological clusters number** compared to the AD (*F=*12.961, *p<*.001; *ηp²=*.18; [–3.357, –0.957]) and control groups (*F=*19.996, *p<*.001; *ηp²=*.23; [–3.613, –1.382]), as did the PPD group relative to controls (*F=*16.574, *p<*.001; *ηp²=*.25; [–3.570, –1.211]). No significant differences were obtained for the PPD relative to the bvFTD (*F=*0.105, *p=*.747; *ηp²=*.00; [–1.158, 1.602]) and AD groups (*F=*3.951, *p=*.053; *ηp²=*.09; [–3.396, 0.026]), nor for the AD group relative to controls (*F=*1.075, *p=*.304; *ηp²=*.02; [–1.824, 0.578]). Interestingly, no group differences remained after controlling for the number of phonologically evoked words.

No significant group differences were obtained in the **phonological mean cluster size** (bvFTD vs PPD: *F=*4.042, *p=*.051; *ηp²=*.09; [–1.088, 0.002], bvFTD vs AD: *F=*1.725, *p=*.194; *ηp²=*.03; [–0.569, 0.118], bvFTD vs controls: *F=*1.380, *p=*.244; *ηp²=*.02; [–0.551, 0.143], AD vs controls: *F=*0.124, *p=*.726; *ηp²=*.00; [–0.391, 0.274], PPD vs controls: *F=*2.526, *p=*.118; *ηp²=*.05; [–0.088, 0.752]). The only exception was the PPD group which produced significantly more phonological mean cluster size compared to the AD group (*F=*5.854, *p=*.020; *ηp²=*.12; [0.099, 1.095]). The difference between the PPD and AD groups remained significant after controlling for the number of phonologically evoked words.

The bvFTD group showed lower **phonological switches number** compared to the AD (*F=*17.322, *p<*.001; *ηp²=*.23; [–7.326, –2.566]) and control groups (*F=*38.345, *p<*.001; *ηp²=*.37; [–7.625, –3.906]), as did the PPD group relative to the AD (*F=*6.460, *p=*.015; *ηp²=*.13; [–9.006, –1.034]) and control groups (*F=*15.645, *p<*.001; *ηp²=*.24; [–7.288, –2.379]). No significant differences were observed between the bvFTD and PPD groups (*F=*0.017, *p=*.897; *ηp²=*.00; [–2.530, 2.880]), as between the AD and control groups (*F=*0.932, *p=*.338; *ηp²=*.01; [–3.663, 1.276]). Interestingly, no group differences remained after controlling for the number of phonologically evoked words.

The total **frequency of words** produced was significantly lower for the bvFTD group compared to the AD (*F=*10.911, *p=*.002; *ηp²=*.16; [–5482.839, –1343.134]) and control groups (*F=*7.204, *p=*.009; *ηp²=*.10; [–5426.791, –795.509]). No significant differences were observed for the PPD group relative to the bvFTD (*F=*2.047, *p=*.160; *ηp²=*.04; [–3926.274, 667.395]), AD (*F=*0.568, *p=*.455; *ηp²=*.01; [–4710.721; 2147.075]), and control groups (*F=*0.102, *p=*.751; *ηp²=*.00; [–3537.714, 2566.637]), as between the AD and control groups (*F=*0.020, *p=*.888; *ηp²=*.00; [–2969.041, 2575.963]). Interestingly, no group differences remained after controlling for the number of phonologically correct words.

The bvFTD group produced a significantly higher number of **very frequent words** compared to the AD (*F=*10.188, *p=*.002; *ηp²=*.15; [–2.675, –0.612]) and control groups (*F=*10.448, *p=*.002; *ηp²=*.14; [–2.408, –0.569]). No significant differences were obtained for PPD group relative to the bvFTD (*F=*0.872, *p=*.356; *ηp²=*.02; [–1.763, 0.647]), AD (*F=*0.990, *p=*.325; *ηp²=*.02; [–2.392, 0.811]) and control groups (*F=*0.108, *p=*.743; *ηp²=*.00; [–1.325, 0.951]) groups, as for the AD group relative to controls (*F=*0.104, *p=*.748; *ηp²=*.00; [–1.261, 0.910]). However, the PPD groups produced a significantly higher number of very frequent words compared to controls after controlling for the number of phonologically evoked words (*F=*6.815, *p=*.012; *ηp²=*.07; [0.185, 1.62])

The bvFTD group produced a significantly fewer number of **frequent words** compared to the AD (*F=*5.029, *p=*.029; *ηp²=*.08; [–1.715, –0.097]) and control groups (*F=*8.840, *p=*.004; *ηp²=*.12; [–1.809, –0.355]). No significant differences were observed for the PPD group relative to the bvFTD (*F=*2.013, *p=*.163; *ηp²=*.04; [–1.664, 0.290]), AD (*F=*0.054, *p=*.817; *ηp²=*.00; [–1.616, 1.281]), and control groups (*F=*0.001, *p=*.976; *ηp²=*.00; [–1.005, 1.036]), as for the AD group compared to controls (*F=*0.480, *p=*.491; *ηp²=*.01; [–1.278, 0.620]). Interestingly, no group differences remained after controlling the number of phonologically correct words.

The bvFTD group produced a significantly fewer number of **rare words** compared to the AD (*F=*13.236, *p<*.001; *ηp²=*.19; [–2.311, –0.670]) and control groups (*F=*24.115, *p<*.001; *ηp²=*.27; [–2.548, –1.074]), as did the PPD group relative to controls (*F=*7.154, *p=*.010; *ηp²=*.12; [–2.278, –0.325]). No significantly differences were observed for the PPD group compared to the bvFTD (*F=*2.079, *p=*.157; *ηp²=*.05; [–1.413, 0.235]) and AD groups (*F=*3.636, *p=*.063; *ηp²=*.08; [–2.631, 0.074]), as for the AD group compared to controls (*F=*0.158, *p=*.693; *ηp²=*.00; [–1.181, 0.789]). Interestingly, no group differences remained after controlling the number of phonologically correct words.

All clinical groups produced significantly fewer **very rare words** compared to controls, including bvFTD (*F=*25.762, *p<*.001; *ηp²=*.29; [–7.025, –3.057]), AD (*F=*11.740, *p=*.001; *ηp²=*.15; [–5.390, –1.420]), and PPD groups (*F=*25.801, *p<*.001; *ηp²=*.34; [–6.834, –2.962]). No significant differences were observed between clinical groups (bvFTD vs PPD: *F=*0.023, *p=*.880; *ηp²=*.00; [–2.579, 2.218], bvFTD vs AD: *F=*2.790, *p=*.100; *ηp²=*.05; [–3.683, 0.334], AD vs PPD: *F=*1.415, *p=*.241; *ηp²=*.03; [–4.140, 1.068]). However, the bvFTD group produced significantly higher very rare words compared to the AD group after controlling for the number of phonologically correct words (*F=*4.772, *p=*.033; *ηp²=*.02; [0.040, 1.24]). The significant difference between the PPD and control groups remained after controlling the number of semantically correct words.

**Discursive level**

Regarding the Anamnestic interview and Narrative speech tasks’ subscores, because speech-therapists had to rate lexicon, syntax, informativity, and pragmatism for both tasks, we grouped them to avoid redundancy and to reduce intra-individual variability. Basically, all the scores for both tasks were converted to percentages of maximum possible (POMP). POMPs were then averaged when they were rated across both tasks. Results are presented in **Supplementary Table 4**.

The bvFTD group demonstrated a lower score on **fluency** compared to the PPD (*F=*5.071, *p=*.029; *ηp²=*.09; [–25.348, –1.448]), AD (*F=*7.112, *p=*.010; *ηp²=*.11; [–21.994, –3.137]) and control groups (*F=*27.797, *p<*.001; *ηp²=*.29; [–25.836, –11.652]), as did the AD and PPD groups compared to controls (AD: *F=*8.853, *p=*.004; *ηp²=*.12; [–10.296, –2.027], PPD: *F=*9.719, *p=*.003; *ηp²=*.15; [–9.405, –2.048]). No significant difference was observed between AD and PPD groups (*F=*0.028, *p=*.868; *ηp²=*.00; [–7.833, 9.252]).

The bvFTD and AD groups demonstrated a lower score on **fluidity** compared to controls (bvFTD: *F=*7.335, *p=*.009; *ηp²=*.10; [–13.708, –2.079], AD: *F=*6.764, *p=*.011; *ηp²=*.09; [–10.968, –1.441]). No significant differences were observed for the bvFTD group compared to the PPD (*F=*1.663, *p=*.203; *ηp²=*.03; [–15.654, 3.413]) and AD groups (*F=*0.033, *p=*.856; *ηp²=*.00; [–8.742, 7.286]), as for the PPD group compared to the AD (*F=*0.370, *p=*.546; *ηp²=*.01; [–6.239, 11.642]) and control groups (*F=*2.591, *p=*.113; *ηp²=*.04; [–7.112, 0.773]).

The bvFTD group exhibited a lower score on **lexicon** compared to the PPD (*F=*4.546, *p=*.038; *ηp²=*.08; [–16.172, –0.483]) and control groups (*F=*15.271, *p<*.001; *ηp²=*.18; [–14.463, –4.687]), as did the AD group compared to controls (*F=*14.326, *p<*.001; *ηp²=*.18; [–15.087, –4.667]). No significant differences were obtained between bvFTD and AD groups (*F=*0.059, *p=*.809; *ηp²=*.00; [–6.611, 8.435]), as for the PPD group compared to the AD (*F=*2.199, *p=*.145; *ηp²=*.04; [–2.328, 15.386]) and control groups (*F=*1.862, *p=*.178; *ηp²=*.03; [–4.935, 0.935]).

The bvFTD group demonstrated a lower score on **syntax** compared to the AD (*F=*9.986, *p=*.002; *ηp²=*.14; [–18.252, –4.099]) and control groups (*F=*6.335, *p=*.014; *ηp²=*.08; [–14.405, –1.667]). No significant differences were observed between the bvFTD and PPD groups(*F=*2.734, *p=*.105; *ηp²=*.05; [–17.351, 1.683]), as for the AD group compared to the PPD (*F=*3.634, *p=*.063; *ηp²=*.07; [–0.354, 13.155]) and control groups (*F=*1.231, *p=*.271; *ηp²=*.02; [–2.069, –7.243]), and the PPD group compared to controls (*F=*1.158, *p=*.287; *ηp²=*.02; [–2.311, 7.679]).

No significant differences were obtained on **comprehension** for all groups (bvFTD vs PPD: *F=*3.818, *p=*.056; *ηp²=*.07; [–11.783, 0.162], bvFTD vs AD: *F=*2.809, *p=*.099; *ηp²=*.04; [–9.227, 0.816], AD vs PPD: *F=*0.832, *p=*.366; *ηp²=*.02; [–5.321, 2.001], AD vs controls: *F=*3.075, *p=*.084; *ηp²=*.04; [–4.096, 0.265], PPD vs controls (as both performances were equal to 100%, No significant differences could be observed). The only exception was the bvFTD group which exhibited a lower score on comprehension compared to controls (*F=*9.548, *p=*.003; *ηp²=*.12; [–9.807, –2.112]).

The bvFTD group demonstrated a lower score on **informativity** compared to the AD (*F=*4.419, *p=*.040; *ηp²=*.07; [–18.206, –0.449]) and control groups (*F=*45.269, *p<*.001; *ηp²=*.40; [–27.760, –15.063]), as did the AD (*F=*11.640, *p=*.001; *ηp²=*.15; [–15.623, –4.088]) and PPD groups (*F=*18.467, *p<*.001; *ηp²=*.25; [–18.079, –6.586]) compared to controls. No significant differences were retrieved for the PPD group compared to the bvFTD (*F=*3.852, *p=*.055; *ηp²=*.07; [–0.258, 22.343]) and AD groups (*F=*0.479, *p=*.492; *ηp²=*.01; [–15.784, 7.702]).

No significant differences were obtained on **prosody** score for all groups (bvFTD vs PPD: *F=*0.003, *p=*.958; *ηp²=*.00; [–7.049, 6.684], bvFTD vs AD: *F=*1.443, *p=*.234; *ηp²=*.02; [–8.194, 2.047], bvFTD vs controls: *F=*3.238, *p=*.076; *ηp²=*.04; [–7.578, 0.390], AD vs PPD: *F=*0.294, *p=*.590; *ηp²=*.01; [–3.865, 6.715], AD vs controls: *F=*0.869, *p=*.355; *ηp²=*.01; [–3.717, 1.351]). The only exception was the PPD group which exhibited a lower score on prosody compared to controls (*F=*4.953, *p=*.030; *ηp²=*.08; [–6.407, –0.338]).

No significant differences were observed on **intelligibility** score for all groups (bvFTD vs PPD: (*F=*0.007, *p=*.936; *ηp²=*.00; [–3.111, 2.870]), bvFTD vs AD: *F=*1.364, *p=*.248; *ηp²=*.02; [–2.885, 0.759], bvFTD vs controls: *F=*0.839, *p=*.363; *ηp²=*.01; [–2.836, 1.051], AD vs PPD: *F=*2.176, *p=*.147; *ηp²=*.04; [–0.577, 3.747], AD vs controls: *F=*0.031, *p=*.861; *ηp²=*.00; [–1.176, 1.403], PPD vs controls: *F=*0.088, *p=*.768; *ηp²=*.00; [–2.345, 1.740]).

The bvFTD group demonstrated a lower score on **pragmatism** compared to the PPD (*F=*10.733, *p=*.002; *ηp²=*.18; [–27.022, –6.481]), AD (*F=*12.906, *p<*.001; *ηp²=*.18; [–20.236, –5.758]) and control groups (*F=*32.620, *p<*.001; *ηp²=*.32; [–23.243, –11.209]). No significant differences were retrieved for the AD group compared to the PPD (*F=*1.213, *p=*.276; *ηp²=*.03; [–2.782, 9.514]) and control groups (*F=*2.213, *p=*.142; *ηp²=*.03; [–2.845, 0.416]), as for the PPD group compared to controls (*F=*3.380, *p=*.071; *ηp²=*.06; [–7.066, 0.302]).

The bvFTD group exhibited a lower score on **attention** compared to the AD (*F=*5.124, *p=*.027; *ηp²=*.08; [–14.452, –0.890]) and control groups (*F=*12.656, *p<*.001; *ηp²=*.15; [–14.458, –4.069]). No significant differences were observed for the PPD group compared to the bvFTD (*F=*3.633, *p=*.062; *ηp²=*.07; [–0.420, 16.020]), AD (*F=*0.008, *p=*.929; *ηp²=*.00; [–5.298, 5.793]), and control groups (*F=*1.470, *p=*.230; *ηp²=*.03; [–2.319, 0.570]), as for the AD group compared to controls (*F=*1.523, *p=*.222; *ηp²=*.02; [–4.951, 1.168]).

The bvFTD group demonstrated a lower score on **actions** compared to the AD (*F=*6.531, *p=*.013; *ηp²=*.10; [–30.992, –3.772]) and control groups (*F=*9.834, *p=*.003; *ηp²=*.13; [–27.675, –6.154]), as did the PPD group compared to the AD group (*F=*7.958, *p=*.007; *ηp²=*.14; 95% CI [–41.643, –6.973]). No significant differences were retrieved for the PPD group compared to the bvFTD (*F=*1.369, *p=*.248; *ηp²=*.03; [–7.019, 26.603]) and control groups (*F=*0.848, *p=*.361; *ηp²=*.01; [–15.737, 5.824]), as for the AD group compared to controls (*F=*0.328, *p=*.569; *ηp²=*.00; [–8.116, 14.649]).

The bvFTD group exhibited a lower score on **narrative** compared to the PPD (*F=*6.315, *p=*.015; *ηp²=*.11; [–21.198, –2.364]), AD (*F=*14.126, *p<*.001; *ηp²=*.19; [–22.672, –6.918]) and control groups (*F=*16.973, *p<*.001; *ηp²=*.20; [–23.063, –8.014]). No significant differences were observed for the AD group compared to the PPD (*F=*1.735, *p=*.194; *ηp²=*.04; [–3.350, 16.057]) and control groups (*F=*0.000, *p=*.983; *ηp²=*.00; [–7.765, 7.601]), as for the PPD group compared to controls (*F=*0.730, *p=*.396; *ηp²=*.01; [–10.609, 4.262]).

**Supplementary Table 4: Qualitative data for discursive processing**

|  | **Mean ± SD** | | | |
| --- | --- | --- | --- | --- |
| **Sub assessments** | **bvFTD** | **AD** | **PPD** | **Controls** |
| *N* | 33 | 30 | 21 | 40 |
| Fluency (%) | 81.2^#✝*^ ± 21.8 | 94.0^*^ ± 11.9 | 94.3^*^ ± 11.2 | 100 ± 0.00 |
| Fluidity (%) | 90.9^*^ ± 17.4 | 92.0^*^ ± 12.4 | 96.2 ± 10.2 | 99.0 ± 4.41 |
| Lexicon (%) | 88.8^#*^ ± 14.3 | 87.4^*^ ± 14.1 | 97.1 ± 7.17 | 98.8 ± 4.04 |
| Syntax (%) | 84.5^✝*^ ± 16.8 | 96.2 ± 8.06 | 91.9 ± 10.3 | 91.0 ± 9.82 |
| Comprehension (%) | 93.9^*^ ± 11.7 | 98.0 ± 6.10 | 100 ± 0.00 | 100 ± 0.00 |
| Informativity (%) | 77.6^✝*^ ± 18.4 | 87.6^*^ ± 15.1 | 87.1^*^ ± 16.8 | 98.5 ± 5.805 |
| Prosody (%) | 95.2 ± 12.3 | 98.0 ± 6.10 | 96.2^*^ ± 8.05 | 99.5 ± 3.16 |
| Intelligibility (%) | 98.8 ± 4.85 | 100 ± 0.00 | 99.0 ± 4.36 | 99.5 ± 3.16 |
| Pragmatism (%) | 83.9^#✝*^ ± 18.7 | 98.3 ± 4.61 | 96.7 ± 11.1 | 100 ± 0.00 |
| Attention (%) | 90.3^✝*^ ± 15.9 | 97.3 ± 8.68 | 99.0 ± 4.36 | 100 ± 0.00 |
| Actions (%) | 58.8^✝*^ ± 26.9 | 75.8 ± 25.2 | 65.7^✝^ ± 26.9 | 72.5 ± 20.6 |
| Narrative (%) | 74.5^#✝*^ ± 16.0 | 88.6 ± 13.6 | 86.7 ± 11.5 | 90.0 ± 15.0 |

Abbreviations*:* SD = standard deviation; bvFTD = behavioral variant frontotemporal degeneration; PPD = primary psychiatric disorder; AD = Alzheimer’s disease; *N* = sample size; % = percentage; ^*^vs controls (p < .05), ^#^vs PPD (p < .05), ^✝^vs AD (p < .05).

**2.3 Neuroimaging correlations**

Analyses were performed with 5000 permutations and familywise error correction (FWE, *p* < 0.05). Clusterwise significance was determined with threshold-free cluster enhancement (TFCE)^48,49^, and only clusters >100 voxels are reported. Results are organized below according to each group rather than level, in Supplementary Tables 5, 6 and 7.

**Supplementary Table 5: Imaging correlations (FSL) for the bvFTD group (N=22)**

|  |  |  | **FSL coordinates of the maximum intensity voxel** | | | **Regional label for coordinated** | |
| --- | --- | --- | --- | --- | --- | --- | --- |
| **Composites** | **Clusters (voxels)** | **MAX** | **X** | **Y** | **Z** | | **MAX region** |
| **Lexical** | 1549 | 0.973 | 28 | 44 | 16 | | Right cerebellum, posterior lobe |
|  | 690 | 0.967 | 55 | 43 | 13 | | Left cerebellum, posterior lobe |
|  | 128 | 0.957 | 42 | 30 | 19 | | Right cerebellum, posterior lobe (Uvula of Vermis) |
|  | 110 | 0.972 | 76 | 65 | 43 | | Left precentral gyrus |
| **Syntactic** | none |  |  |  |  | |  |
| **Discursive** | 2252 | 0.981 | 62 | 20 | 49 | | Left occipital pole |
|  | 372 | 0.96 | 74 | 64 | 40 | | Left precentral gyrus |
|  | 313 | 0.996 | 63 | 24 | 29 | | Left occipital pole |
|  | 133 | 0.958 | 47 | 94 | 48 | | Left frontal pole (superior frontal gyrus) |
|  | 118 | 0.956 | 36 | 36 | 65 | | Right parietal lobe (precuneus) |
| **Transposing & transcoding** | 56693 | 0.999 | 53 | 45 | 72 | | Left parietal lobe (postcentral gyrus) |

**Supplementary Table 6: Imaging correlations (FSL) for the AD group (N=15)**

|  |  |  | **FSL coordinates of the maximum intensity voxel** | | | **Regional label for coordinated** | |
| --- | --- | --- | --- | --- | --- | --- | --- |
| **Composites** | **Clusters (voxels)** | **MAX** | **X** | **Y** | **Z** | | **MAX region** |
| **Lexical** | 2222 | 0.993 | 49 | 60 | 73 | | Left superior frontal gyrus |
|  | 890 | 0.985 | 60 | 56 | 16 | | Left fusiform cortex |
|  | 866 | 0.991 | 73 | 60 | 26 | | Left middle temporal gyrus |
|  | 628 | 0.990 | 20 | 70 | 16 | | Right temporal pole |
| **Syntactic** | 4607 | 0.999 | 52 | 70 | 70 | | Left superior frontal gyrus |
|  | 3990 | 1 | 23 | 68 | 13 | | Right temporal pole |
|  | 3093 | 0.998 | 64 | 64 | 16 | | Left fusiform cortex |
|  | 266 | 0.971 | 20 | 65 | 50 | | Right precentral gyrus |
| **Discursive** | 3356 | 0.997 | 76 | 56 | 33 | | Left superior temporal gyrus |
|  | 3270 | 0.995 | 19 | 59 | 23 | | Right superior temporal gyrus |
|  | 1040 | 0.986 | 34 | 61 | 64 | | Right superior frontal gyrus |
|  | 739 | 0.984 | 24 | 80 | 44 | | Right middle frontal gyrus |
|  | 714 | 0.993 | 54 | 70 | 64 | | Right superior temporal gyrus |
|  | 694 | 0.986 | 68 | 73 | 38 | | Left frontal operculum |
|  | 571 | 0.977 | 14 | 50 | 36 | | Right superior temporal gyrus |
|  | 392 | 0.975 | 19 | 59 | 49 | | Right postcentral gyrus |
| **Transposing & transcoding** | 16015 | 0.999 | 43 | 81 | 52 | | Right paracingulate gyrus |

**Supplementary Table 7: Imaging correlations (FSL) for the PPD group (N=12)**

|  |  |  | **FSL coordinates of the maximum intensity voxel** | | | **Regional label for coordinated** | |
| --- | --- | --- | --- | --- | --- | --- | --- |
| **Composites** | **Clusters (voxels)** | **MAX** | **X** | **Y** | **Z** | | **MAX region** |
| **Lexical** | none |  |  |  |  | |  |
| **Syntactic** | none |  |  |  |  | |  |
| **Discursive** | 3196 | 0.992 | 31 | 44 | 35 | | Right temporal lobe (parahippocampal gyrus) |
|  | 1102 | 0.968 | 52 | 34 | 53 | | Left parietal lobe (precuneus) |
|  | 697 | 0.966 | 63 | 46 | 33 | | Left temporal lobe (parahippocampal gyrus) |
|  | 289 | 0.962 | 42 | 15 | 36 | | Right lingual gyrus |
|  | 136 | 0.97 | 26 | 32 | 54 | | Right parietal lobe (precuneus) |
| **Transposing & transcoding** | 348 | 0.972 | 45 | 36 | 62 | | Left parietal lobe (precuneus |
|  | 105 | 0.96 | 51 | 35 | 51 | | Left parietal lobe (cingulate gyrus) |

**Supplementary references**

1. Troyer AK, Moscovitch M, Winocur G. Clustering and switching as two components of verbal fluency: evidence from younger and older healthy adults. *Neuropsychology*. 1997;11(1):138-146. doi:10.1037//0894-4105.11.1.138

2. Ledoux K, Vannorsdall TD, Pickett EJ, Bosley LV, Gordon B, Schretlen DJ. Capturing additional information about the organization of entries in the lexicon from verbal fluency productions. *J Clin Exp Neuropsychol*. 2014;36(2):205-220. doi:10.1080/13803395.2013.878689

3. Cintoli S, Favilli L, Morganti R, Siciliano G, Ceravolo R, Tognoni G. Verbal fluency patterns associated with the amnestic conversion from mild cognitive impairment to dementia. *Sci Rep*. 2024;14(1):2029. doi:10.1038/s41598-024-52562-x

4. New B, Pallier C, Brysbaert M, Ferrand L. Lexique 2 : A new French lexical database. *Behavior Research Methods, Instruments, & Computers*. 2004;36(3):516-524. doi:10.3758/BF03195598

5. New B, Brysbaert M, Veronis J, Pallier C. The use of film subtitles to estimate word frequencies. *Applied Psycholinguistics*. 2007;28(4):661-677. doi:10.1017/S014271640707035X

6. Lubineau M, Watkins CP, Glasel H, Dehaene S. Examining the Impact of Reading Fluency on Lexical Decision Results in French 6th Graders. *Open Mind (Camb)*. 2024;8:535-557. doi:10.1162/opmi_a_00140

7. Ashburner J, Friston KJ. Voxel-based morphometry--the methods. *Neuroimage*. 2000;11(6 Pt 1):805-821. doi:10.1006/nimg.2000.0582

8. Good CD, Johnsrude IS, Ashburner J, Henson RNA, Friston KJ, Frackowiak RSJ. A Voxel-Based Morphometric Study of Ageing in 465 Normal Adult Human Brains. *NeuroImage*. 2001;14(1):21-36. doi:10.1006/nimg.2001.0786

9. Smith SM, Jenkinson M, Woolrich MW, et al. Advances in functional and structural MR image analysis and implementation as FSL. *NeuroImage*. 2004;23(SUPPL. 1):S208-S219. doi:10.1016/j.neuroimage.2004.07.051

10. Zhang Y, Brady M, Smith S. Segmentation of brain MR images through a hidden Markov random field model and the expectation-maximization algorithm. *IEEE Trans Med Imaging*. 2001;20(1):45-57. doi:10.1109/42.906424

11. Andersson J, Jenkinson M, Andersson J. Non-linear optimisation FMRIB Technial Report TR07JA1. In: 2007. Accessed February 4, 2025. https://www.semanticscholar.org/paper/Non-linear-optimisation-FMRIB-Technial-Report-TR-07-Andersson-Jenkinson/7018dff7b9e2d05f2b43a0fe8b0a3598e2f213f2

12. Andersson J, Jenkinson M, Andersson J. Non-linear registration aka Spatial normalisation FMRIB Technical Report TR07JA2. In: 2007. Accessed February 4, 2025. https://www.fmrib.ox.ac.uk/datasets/techrep/tr07ja2/tr07ja2.pdf

13. Rueckert D, Sonoda LI, Hayes C, Hill DL, Leach MO, Hawkes DJ. Nonrigid registration using free-form deformations: application to breast MR images. *IEEE Trans Med Imaging*. 1999;18(8):712-721. doi:10.1109/42.796284

14. Good CD, Scahill RI, Fox NC, et al. Automatic differentiation of anatomical patterns in the human brain: validation with studies of degenerative dementias. *Neuroimage*. 2002;17(1):29-46. doi:10.1006/nimg.2002.1202
